# Cellulose photonic pigments

**DOI:** 10.1038/s41467-022-31079-9

**Published:** 2022-06-13

**Authors:** Richard M. Parker, Tianheng H. Zhao, Bruno Frka-Petesic, Silvia Vignolini

**Affiliations:** Yusuf Hamied Department of Chemistry, University of Cambridge, Lensfield Road, Cambridge, CB2 1EW UK

**Keywords:** Bioinspired materials, Colloids, Liquid crystals, Photonic crystals, Self-assembly

## Abstract

When pursuing sustainable approaches to fabricate photonic structures, nature can be used as a source of inspiration for both the nanoarchitecture and the constituent materials. Although several biomaterials have been promised as suitable candidates for photonic materials and pigments, their fabrication processes have been limited to the small to medium-scale production of films. Here, by employing a substrate-free process, structurally coloured microparticles are produced via the confined self-assembly of a cholesteric cellulose nanocrystal (CNC) suspension within emulsified microdroplets. Upon drying, the droplets undergo multiple buckling events, which allow for greater contraction of the nanostructure than predicted for a spherical geometry. This buckling, combined with a solvent or thermal post-treatment, enables the production of dispersions of vibrant red, green, and blue cellulose photonic pigments. The hierarchical structure of these pigments enables the deposition of coatings with angular independent colour, offering a consistent visual appearance across a wide range of viewing angles.

## Abstract (web summary)

Several biomaterials have been promised as suitable candidates for photonic materials and pigments, but their fabrication processes have been limited to the small to medium-scale production of films. Here, the authors demonstrate a substrate-free process to fabricate structurally coloured microparticles via the confined self-assembly of a cholesteric cellulose nanocrystal (CNC) suspension within emulsified microdroplets.

## Introduction

Colour is an essential form of communication in our society, with the production and use of dyes and pigments dating back to prehistoric times. In the last few decades, the available colour palette has been enlarged and diversified enormously by the development of so-called interference or effect pigments^1,2^. While such photonic pigments are increasingly found in a wide variety of consumer products, spanning from automotive paints and security inks to textiles, cosmetics and food, they often rely on the use of energy-intensive methods, synthetic polymers^3^ or inorganic materials (whose extraction raises ethical concerns^4^). When considering sustainable approaches to photonic pigments, nature can be used as a source of inspiration in terms of both the nanoarchitecture and the constituent materials^5–7^. Inspired by the hierarchical arrangement of cellulose found in structurally coloured plants^8^, here we report a substrate-free, emulsion-based methodology to produce cellulosic photonic pigments with vibrant colour spanning across the entire visible spectrum.

It is well known that colloidal suspensions of cellulose nanocrystals (CNCs) spontaneously self-organise above a certain volume fraction into a cholesteric (i.e., chiral nematic) colloidal liquid crystal, whereby the individual nanoparticles align locally along a helicoidal structure^9,10^. Moreover, evaporating a cholesteric CNC suspension can lead to photonic films that display vibrant, iridescent colour^10–12^. However, the self-assembly of CNCs confined within a spherical geometry, such as that of an emulsified droplet, has failed to produce microparticles with structural colour in the visible range, despite the attempts of several research groups^13–19^. The colour of CNC-based photonic materials is defined by the periodicity of the underlying helicoidal nanoarchitecture, referred to as the ‘pitch’, $p$. In general, the value of the pitch is affected by suspension-related parameters (e.g. pH or ionic strength) and, once the suspension is kinetically arrested^10^, by the geometry in which self-assembly takes place. We previously reported that for a hierarchical spherical geometry undergoing isotropic contraction, the relationship that regulates the pitch with the CNC volume fraction scales as $p\propto {\phi }^{-1/3}$, instead of $p\propto {\phi }^{-1},$ as expected for vertically aligned domains in a drying film^12,20^. This reduced compression leads to microparticles with pitch values in the micron-range, correlating to reflection in the infrared regime rather than at visible wavelengths. In this study, the inherent limitation of CNC self-assembly under spherical confinement is overcome by exploiting the interfacial buckling of kinetically arrested microdroplets of an aqueous CNC suspension. This emulsion-based methodology combines a careful optimisation of the aqueous CNC suspension with the controlled removal of water from the buckled CNC microparticles, resulting in vibrant red, green, and blue dispersions of cellulosic photonic pigments. Furthermore, we show that the hierarchical structure of these pigments enables the production of coatings with angular independent structural colour.

## Results

### Fabrication of structurally coloured CNC microparticles

**Fig. 1:**
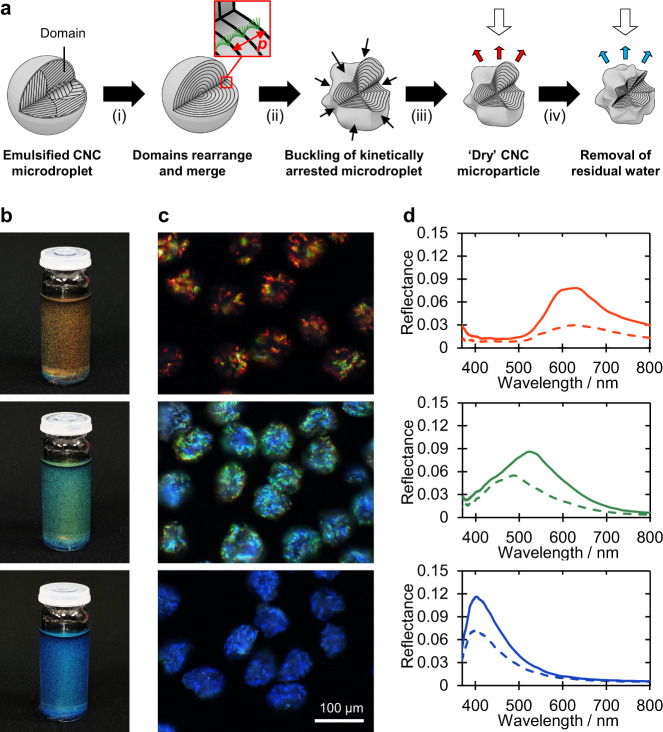
Photonic pigments via the confined self-assembly of cellulose nanocrystals. **a** Schematic summarising the preparation of photonic microparticles: (i) the aqueous CNC suspension was emulsified in hexadecane via a microfluidic flow-focusing device, upon which the cholesteric domains self-organise and merge into a radially aligned monodomain (Frank–Pryce structure); (ii) at a later stage of drying, the microdroplet becomes kinetically arrested and buckles; (iii) once drying is complete, CNC microparticles are formed with a complex surface morphology and visible red colouration; (iv) controlled removal of residual water within the microparticle induces additional buckling resulting in a further blueshift, enabling a full spectrum of photonic microparticles to be produced. **b** Photographs showing red, green, and blue cellulosic photonic pigments suspended at ca. 3.5 mg mL^−1^ in ethyl cinnamate (*n* = 1.56). The vial is 22 mm in diameter. **c** Dark-field microscopy images of individual CNC microparticles in refractive index oil (*n* = 1.55) and (**d**) associated micro-spectra of individual microparticles, collected through left-handed circularly polarisation (LCP, solid line) and right-handed circularly polarisation (RCP, dashed line) filters. The spectra are normalised against a white Lambertian diffuser coated with the same refractive index oil.

In order to produce CNC microparticles with visible colour, an aqueous CNC formulation was first identified that yielded the smallest obtainable pitch when independent of any geometric constraints. It was found that films cast from the anisotropic phase of a commercial CNC suspension (Univ. Maine, 7.0 wt.%), in the presence of additional salt ([NaCl]/[CNC] = 100 µmol g^−1^), have an average pitch of 141 ± 9 nm, which results in reflection in the ultraviolet region (Supplementary Fig. 1). As summarised in Fig. 1a, the same aqueous CNC suspension was then emulsified in hexadecane via a flow-focusing microfluidic device to produce monodisperse water-in-oil microdroplets (Ø ≈ 160 μm). Upon controlled, slow drying beneath a hexadecane layer, the randomly oriented cholesteric CNC domains inside each microdroplet merged and reorganised to form a radially aligned Frank–Pryce monodomain structure (Supplementary Video 1 and Supplementary Fig. 12)^13,20,21^. Further water loss triggered the onset of kinetic arrest, with the periphery of the droplet arresting first due to a radial concentration gradient within the droplet that arose from the evaporative flux at the interface. The earlier arrest at the droplet interface contributed to the formation of a shell that then buckled due to the interplay between compressive capillary forces (that shrink both the droplet radius and surface area as the droplet dries) and the mechanical resistance of the solidifying cholesteric CNC shell to a reduction in its total surface area^22–25^. Significant buckling of the radially aligned cholesteric shell enhanced the pitch compression within the arrested droplet, allowing the inherent limit of the spherical geometry to be overcome and resulting in microparticles that display visible colour.

The initial buckling resulted in red-coloured microparticles (Ø ≈ 100 μm), however additional compression of the pitch was required to produce the dispersions of green and blue CNC microparticles shown in Fig. 1b. This was controllably achieved by applying a thermal or solvent post-treatment to further collapse and buckle the microparticles, as summarised in Supplementary Fig. 2 and discussed in detail later. The irregular and buckled surface of the microparticles can be visualised by dark-field microscopy (Fig. 1c). Unlike concentrically ordered photonic microspheres^26–28^, the majority of the reflected light is at oblique angles, providing a less directional optical response^29^, as evidenced by the microparticles appearing less vibrant in bright-field microscopy (Supplementary Fig. 3). Micro-spectroscopy of individual CNC microparticles confirmed that the colour arises from a single reflection peak that is predominantly left-circularly polarised (Fig. 1d), validating the underlying helicoidal architecture is the same as observed for photonic CNC films.

### Accessing additional pitch compression via desiccation

**Fig. 2:**
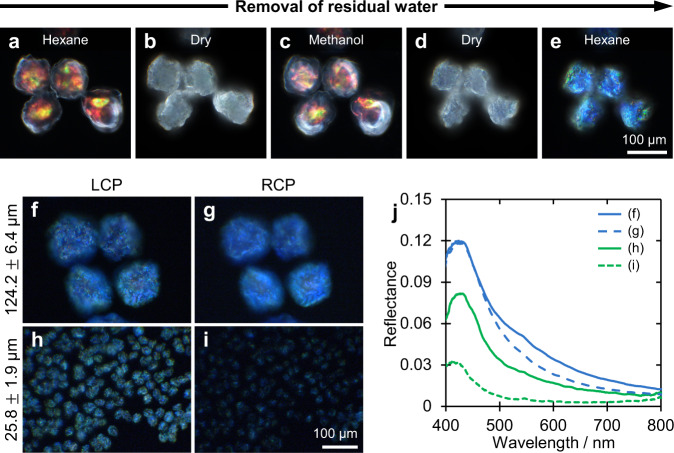
Optical analysis of CNC microparticles during solvent post-treatment. **a**–**e** Sequence of dark-field micrographs showing the decrease in size and the blueshift in optical appearance after removal of residual water by treatment with methanol. The loss of colour in the dry state arises from strong scattering at the microparticle–air interface. **f**–**i** Comparison of the optical appearance of large (ca. 124 μm) and small (ca. 25 μm) CNC microparticles in refractive index oil (*n* = 1.55) when imaged through left-circular polarisation (LCP) and right-circular polarisation (RCP) filters; and (**j**) corresponding micro-spectra of individual microparticles, through LCP (solid line) and RCP (dashed line) filters and normalised against a white Lambertian diffuser coated with the same refractive index oil.

The change in the visual appearance of the CNC microparticles arising from the additional compression upon treatment with a polar solvent is exemplified in Fig. 2. The dried microparticles were first washed with *n-*hexane to remove residual non-volatile hexadecane and surfactant. In this non-polar solvent, the microparticles do not swell and a predominately red colour was reflected (Fig. 2a). When the *n-*hexane was subsequently evaporated, the microparticles appeared white due to strong surface scattering at the air–particle interface, but without a noticeable volume change (Fig. 2b). The CNC microparticles were then immersed in methanol, leading to a small degree of swelling and the reappearance of the red colour due to a reduced refractive index contrast at the particle-solvent interface (Fig. 2c). However, upon evaporation of methanol, the microparticles experienced a significant volume reduction and consequently a pitch compression (Fig. 2d), such that subsequent re-immersion in *n-*hexane revealed blue colouration (Fig. 2e). Less polar solvents can be used to induce smaller volume contractions that result in intermediate colours, with the green CNC microparticles shown in Fig. 1 obtained with isopropanol. Notably, while repeated washing with isopropanol does not induce any further colour change, exposing the isopropanol-washed microparticles to a more polar solvent (e.g., methanol) results in a further irreversible blueshift (Supplementary Fig. 4).

The effectiveness of the methanol treatment on different sized microparticles was tested. It was found that although microparticles of three sizes (∅ ≈ 26, 80, 124 μm) all demonstrated a similar blue appearance after methanol treatment, the circular polarisation of the optical response differed (Supplementary Fig. 5). While the smallest microparticles reflected predominantly left-circularly polarised light, similar to conventional drop-cast CNC films^30^, the largest microparticles showed comparable intensity in both circular polarisations (Fig. 2f–j). This trend in polarisation response can be attributed to the distortion of the helicoidal architecture within the buckled microparticles. First, the local shear of the cholesteric domains causes a distortion of the CNC helicoidal arrangement, which results in the reflected light having an elliptical polarisation that is still predominantly left-handed, i.e. that can be decomposed in a main left-circularly polarised (LCP) component as well as a small right-circularly polarised (RCP) component^12,31^. Second, at a larger scale the buckling of the interface results in highly tilted cholesteric regions, effectively acting as birefringent retardation plates. This enables the conversion of incident RCP light into LCP light, which can be reflected by deeper cholesteric regions within the microparticles, and then converted back into RCP light^32,33^. These effects are expected to be more prevalent in larger microparticles since they are both thicker and more buckled (Supplementary Fig. 6).

**Fig. 3:**
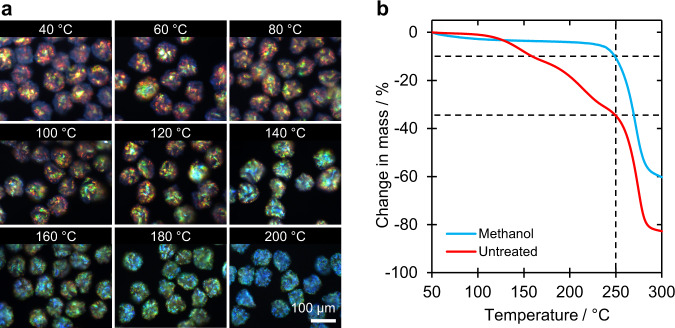
The impact of residual water on the optical appearance of CNC microparticles. **a** Dark-field micrographs showing the effect of thermal treatment at different temperatures on the reflected colour of *n*-hexane washed CNC microparticles. **b** Thermogravimetric analysis (TGA) from 50 to 300 °C for a sample of CNC microparticles with, and without, treatment with methanol. The marked difference in the two curves is attributed to the removal of almost all the residual solvent trapped within the CNC microparticles.

Given that methanol and isopropanol are able to form hydrogen bonds with water and thus act as dehydrants (e.g. methanol is routinely used to fix proteins^34,35^), the volume contraction and corresponding pitch reduction induced by washing the ‘dry’ CNC microparticles with a polar solvent was attributed to the removal of residual water. This was verified by studying the effect of thermal treatment on the hexane-washed microparticles. Specifically, the microparticles were exposed to a constant temperature chosen between 40 and 200 °C for sixty minutes in a dry nitrogen atmosphere (Supplementary Fig. 7), followed by optical microscopy in refractive index-matching oil (*n* = 1.55) at ambient temperature (Fig. 3a). Despite an apparent linear decrease in diameter with temperature, the CNC microparticles heated below 100 °C present only minor colour change suggesting this initial volume contraction had little impact on the buckled surface (Supplementary Fig. 8a). However, samples exposed to temperatures above 100 °C displayed a more significant blueshift, with microparticles heated at 200 °C exhibiting a blue appearance comparable to their methanol-washed analogues (Supplementary Fig. 8b and Fig. 1d). Importantly, while the solvent-treated CNC microparticles disintegrate upon immersion in water, the microparticles heated at 200 °C maintain their integrity and ability to reflect visible light (with a moderate redshift), possibly as a result of the additional thermal desulfation of the CNCs (Supplementary Fig. 9)^36–39^.

**Fig. 4:**
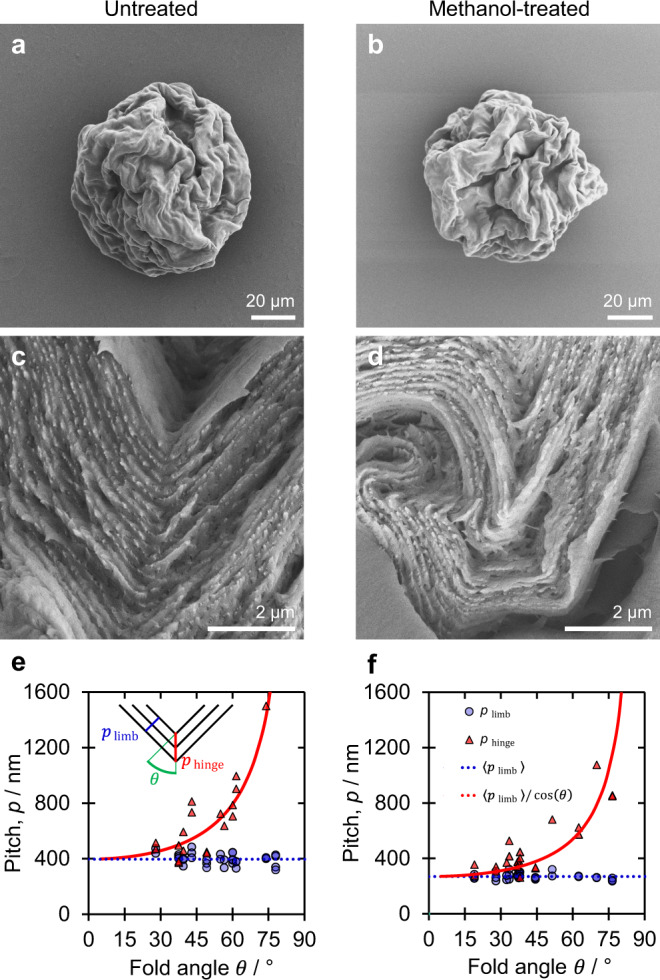
The role of interfacial buckling on the pitch of the radially aligned CNC microparticles. Scanning electron microscopy (SEM) images of a representative microparticle (**a**) prior to and (**b**) after treatment with methanol, showing the volume contraction and increase in buckling. Corresponding cross-sectional images in (**c**) and (**d**) show the related decrease in the pitch of the helicoidal structure. **e**, **f** Limb and hinge pitches of (**e**) hexane-washed and (**f**) methanol-treated microparticles plotted as a function of the fold angle $\theta$, as defined in the inset schematic. For both samples, the limb pitch is independent of $\theta$, while the hinge pitch generally follows the stated trigonometric relationship.

To quantify the amount of residual water in the CNC microparticles, samples with and without methanol treatment were characterised by thermogravimetric analysis (TGA), as reported in Fig. 3b. As expected, both curves exhibit a large mass loss starting at 250 °C, corresponding to the thermal degradation of cellulose^40^. However, prior to reaching this temperature, a significant mass loss (ca. 35%) was recorded in the sample without methanol treatment, while a much smaller mass loss (ca. 10%) was observed for the methanol-treated sample. After correcting for desulfation (see Supplementary Discussion 1), the mass fraction of removable water was calculated to be approximately 30% for the untreated sample. Scanning electron microscopy (SEM) measurement of the highly buckled CNC microparticles showed a decrease in the average diameter from 88.5 ± 0.8 μm to 77.9 ± 0.5 μm upon methanol treatment (Fig. 4a, b and Supplementary Table 1). This corresponds to a 12.1 ± 1.1% diameter decrease, which, given it underestimates the impact of buckling, is in good agreement with the value of 13.9% estimated from the TGA analysis and calculated in Supplementary Discussion 1. Another important indication of the dehydrant effect of methanol is the reduced mass loss upon pyrolysis above 250 °C, which suggests that reaction with water to form CO and CO_2_ no longer occurs (Supplementary Fig. 10)^41^. This further supports our hypothesis that both solvent and thermal treatment cause a blueshift in the reflected colour by the removal of residual water.

As expected from the optical analysis, SEM cross-sections of the microparticles revealed that the cholesteric domain remains locally well-aligned to the highly buckled surface (Fig. 4c, d). The pitch measured in regions away from the hinge of a fold (${p}_{{{{{{\rm{limb}}}}}}}$, which are primarily responsible for the visual appearance) are consistent across multiple regions and microparticles, with the average pitch $\left\langle {p}_{{{{{{\rm{limb}}}}}}}\right\rangle$ reduced from 396 ± 31 nm to 269 ± 16 nm after the methanol treatment, in agreement with the observed optical response (Fig. 4e, f). In contrast, the pitch measured in the hinge of a fold (${p}_{{{{{{\rm{hinge}}}}}}}$, contributing to a negligible volume fraction) was much more variable and depended on the fold tightness, with ${p}_{{{{{{\rm{hinge}}}}}}}\approx \left\langle {p}_{{{{{{\rm{limb}}}}}}}\right\rangle /{{\cos }} \, \theta$, where the fold angle $\theta$ is defined in Fig. 4. Interestingly, the percentage of pitch compression in the limbs is ca. 32%, which matches well with the volume contraction of the microparticle (32.1 ± 1.5%) rather than the reduction in diameter (12.1 ± 1.1%) as estimated from SEM (cf. Fig. 4a, b). This indicates that buckling of the spherical microparticle allows the pitch to be locally compressed more significantly than expected for this geometry^20^. This greater compression, approaching that of a drop-cast film, is what enables reflection at visible wavelengths to be achieved. A multi-step buckling process, as suggested by the SEM images, can explain the mechanism of pitch compression and the route to volume shrinkage. Upon each buckling event during the microparticle drying process, ${p}_{{{{{{\rm{hinge}}}}}}}$ would stop contracting and thus would “record” a transient pitch, while ${{p}}_{{{{{{\rm{limb}}}}}}}$ would continue to shrink uniformly (with a corresponding $\theta$ increase), giving rise to coherent colour across the microparticle. This process would repeat, resulting in additional buckling events, leading to several generations of hinges where the oldest folds have the largest ${p}_{{{{{{\rm{hinge}}}}}}}$ and $\theta$. While buckling events facilitate the removal of water from the microparticles (as described below), they also increase the ability for the resulting distorted structure to withstand greater mechanical stress. In contrast to a drop-cast CNC film, where complete collapse of the cholesteric phase is achieved upon evaporation, this behaviour makes it increasingly difficult for residual water to escape upon drying under ambient conditions. As such the removal of this trapped water requires additional mechanical deformation, achievable either by displacement with a polar solvent or by thermal treatment, to allow capillary forces to further contract the microparticle.

### The role of interfacial buckling in achieving visible colour

**Fig. 5:**
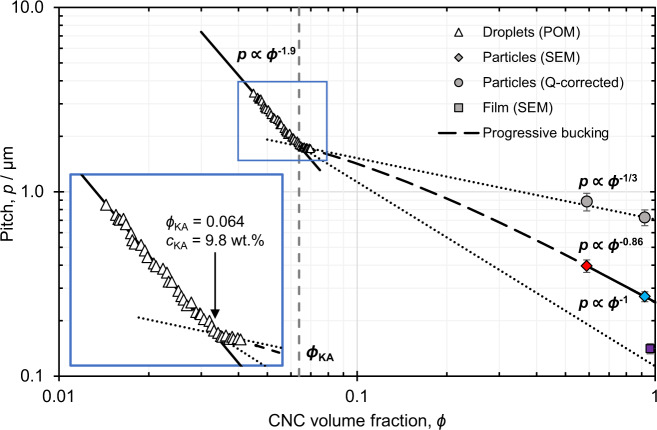
The reduction in the cholesteric pitch upon drying a microdroplet of CNC suspension to form a photonic microparticle. The cholesteric pitch measured in the droplets by polarised optical microscopy (triangles) initially decreases following $p\propto {\phi }^{-1.9}$, until a transition at $\phi$ = 6.4% *v*/*v*, upon which it initially appears to follow $p\propto {\phi }^{-1/3}$ (see inset). An extrapolation of $p\propto {\phi }^{-1}$ from this transition is in reasonable agreement with SEM cross-sectional analysis of the corresponding film (square), validating this is the onset of kinetic arrest (${\phi }_{{{{{{\rm{KA}}}}}}}$). Extrapolation of the $p\propto {\phi }^{-1/3}$, does not match with the pitch measured from the red and blue microparticles (respectively red and blue diamonds), with the additional pitch compression attributed to progressive buckling post kinetic arrest. By considering the isoperimetric quotient $Q$ of the microparticle cross-sections, the pitch they would have if buckling could be prevented can be estimated (circles), which is in good agreement with the $p\propto {\phi }^{-1/3}$ power law, confirming the key role of buckling in causing additional radial pitch contraction upon further compression. Error bars represent the average deviation of ${p}_{{{{{{\rm{limb}}}}}}}$ from SEM cross-sections.

The mechanism leading to visible colour can be clarified by tracking the evolution of the cholesteric pitch within a droplet as it dries to form a buckled microparticle. The relationship between the pitch and the CNC volume fraction (determined from the diameter change) was measured by recording a timelapse of a drying droplet using transmission polarised optical microscopy (Fig. 5a: triangles). A clear transition is observed at 6.4% *v*/*v* (i.e. 9.8 wt.%), which is ascribed to kinetic arrest^42^. Beyond this point the pitch trajectory switches from ${\phi }^{-1.9}$ to ${\phi }^{-1/3}$, the latter being the scaling law expected for a completely arrested Frank–Pryce cholesteric structure in an isotropically shrinking sphere^20^. The pitch of the final untreated and methanol-treated microparticles, as measured in cross-section by SEM, is also plotted (Fig. 5a: diamonds), with the corresponding CNC volume fractions determined from the TGA analysis. These values are clearly smaller than the projection of the ${\phi }^{-1/3}$ scaling law, suggesting that another mechanism intervenes. If this discrepancy can be attributed to buckling, the isoperimetric quotient, $Q=4\pi {\Sigma }_{2}/{{{{{{\mathscr{P}}}}}}}_{2}^{2}$, defined from the cross-section area of the microparticles, ${\Sigma }_{2}$, and their corresponding cross-section perimeter, ${{{{{{\mathscr{P}}}}}}}_{2}$, can be used to quantify it (see Supplementary Discussion 2). From mass conservation arguments, $Q$ (which is smaller than unity for non-circular cross-sections) must be equal to the scaling factor impacting the particle contraction in the radial direction, and thus allows for an estimation of the radial pitch contraction due to buckling. A statistical analysis on both untreated and methanol-treated microparticles estimates $Q$ to be approximately 0.45 and 0.37, respectively (Supplementary Fig. 11). This allows for an estimation of a hypothetical ‘unbuckled pitch’, which is found to follow the previously expected ${\phi }^{-1/3}$ scaling law (Fig. 5a: circles), corroborating this interpretation. As such, the anisotropic buckling of the drying CNC microdroplet allows the power law to evolve from that expected for isotropic spherical compression (${\phi }^{-1/3}$) to one that approaches that of unilateral compression (i.e., ${\phi }^{-0.86}{\to \phi }^{-1}$), as discussed further in Supplementary Discussion 2. This additional pitch contraction is key to accessing visible wavelengths, suggesting that for the production of photonic pigments an early kinetic arrest is vital to allow for the buckling to sufficiently develop during the drying process. By optimising both suspension parameters (e.g., ionic strength) and the cellulose source, the CNC suspension employed in this study becomes kinetically arrested at a sufficiently low volume fraction (6.4% v/*v*). Combining this with the small initial pitch in suspension (facilitated by a steep ${\phi }^{-1.9}$ power law prior to kinetic arrest) and the use of only the fully anisotropic phase (expediting an early radial self-organisation upon emulsification), it allows for visibly coloured CNC microparticles to be achieved^20^. For completeness, a dish-cast film with vertically aligned domains was also prepared from the same CNC suspension (Supplementary Fig. 1), and the pitch was found to agree with the ${\phi }^{-1}$ scaling law expected for the film geometry when assuming the same kinetic arrest transition as for the drying microdroplets^12,20^.

Two different mechanisms can be independently responsible for the anisotropic compression that results in buckling of the kinetically arrested Frank–Pryce structure. First, an arrested cholesteric suspension may preferentially compress along the helical axis upon water loss. Indeed, strong alignment of rigid fibres can lead to anisotropic swelling or contraction that is favoured in the perpendicular direction^43,44^, and to anisotropic Young moduli in the dry state^45^. For the radial helical arrangement of the Frank–Pryce structure, this results in an imbalance of radial to orthoradial compression upon volume contraction^46,47^, leading to an excess of surface area to maintain a spherical shape. Second, the concentration front propagating inwards in the arrested droplet can lead to a more rigid shell that resists orthoradial compression upon further volume contraction^48^. Indeed, buckling of a spherical shell was reported for drying droplets of isotropic colloidal particle suspensions^24^, while it was prevented when high solvent permeability was maintained^49^, suggesting that buckling can be solely driven by the kinetics of water loss. In the latter case, an insufficient orthoradial contraction is compensated by an enhanced radial compression. Regardless of the precise mechanism, the high resistance of the drying microparticle to orthoradial compression prevents volume contraction if the spherical shape is maintained, so buckling is a necessary condition for further water removal. Interestingly, buckling also comes with high deformation costs and makes further volume contraction more difficult. As it contracts, the concentric cholesteric structure undergoes several buckling events resulting in several generations of wrinkles, which are increasingly able to resist further orthoradial compression despite the low water vapour pressure. This explains why capillary forces were not able to completely compress the ‘dry’ microparticles in hexane, while further desiccation with either polar solvent or by heat treatment allowed for further compression and buckling with higher order wrinkles.

**Fig. 6:**
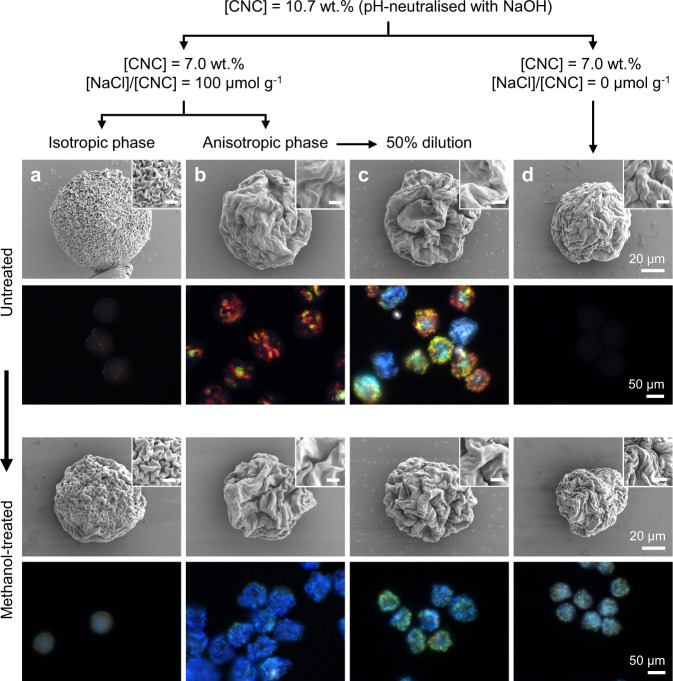
The role of CNC suspension formulation on the morphology and visual appearance of the photonic microparticles. Scanning electron microscope and dark-field optical microscope images of CNC microparticles produced from different formulations of the commercial CNC suspension, and recorded prior to (top), and after methanol treatment (bottom): For the standard conditions employed in this study ([CNC = 7.0 wt.% [NaCl]/[CNC] = 100 μmol g^−1^), the suspension phase separates into (**a**) an isotropic phase and (**b)** an anisotropic phase. Microparticles produced from the anisotropic phase show vibrant colour, while those produced from the isotropic phase are only weakly coloured. **c** Diluting the anisotropic phase suspension in (**b**) by 50 vol.% with water to yield an isotropic suspension prior to emulsification results in vibrantly microparticles with a range of colours, which can be blueshifted upon further solvent treatment. **d** Diluting the same CNC source in the absence of any additional salt ([NaCl]/[CNC] = 0 μmol g^−1^) resulted in a fully anisotropic (i.e. unfractionated) suspension that produced less intense, redshifted microparticles. The SEM inset scalebar is 5 µm.

An important consequence of anisotropic compression and buckling is a steeper pitch decrease than that predicted by the power law for an isotropic contraction ($p\propto {\phi }^{-1/3}$). To understand how the formulation of the initial CNC suspension influences the formation of a Frank–Pryce structure and its evolution into a buckled, coloured microparticle, three variations were prepared and compared against the standard (anisotropic) formulation (Fig. 6), with the self-assembly also monitored over time (Supplementary Figs. 12–15). A 50% dilution of the standard CNC suspension resulted in a more pronounced core-shell morphology within the drying droplets, resulting in irregular, collapsed microparticles with larger-period wrinkles. This morphology led to more inter- and intra-particle variations in colour, however it is notable that all microparticles were blue-green after subsequent methanol treatment. In a second variation, the isotropic fraction of the original CNC suspension was emulsified instead of the anisotropic fraction. The inability of this suspension to assemble into a Frank–Pryce structure within the microdroplet resulted in the formation of more spherical microparticles with only fine surface buckling. Faint structural colour arises from this thin shell, while the disordered, polydomain core leads to significant broadband scattering (i.e., whiteness). In a third variation, electrolytes (i.e., NaCl) were not added to the CNC suspension, resulting in a fully anisotropic and viscous suspension at 7.0 wt.%. Although the resulting microparticles were buckled, weak cyan colour was only apparent after methanol treatment. This redshift in colour was attributed to a larger initial pitch, with the untreated microparticles initially in the infrared, while the lower intensity is explained by the inability of this viscous suspension to fully develop into a well-ordered Frank–Pryce arrangement prior to becoming kinetically trapped. The origin of these various buckling morphologies and how they lead to such diversity in optical response is further discussed in Supplementary Discussion 3, with the self-assembly pathway summarised schematically in Supplementary Fig. 16. Importantly, these results affirm that the anisotropy of the cholesteric contraction is important in the evolution of the buckled microparticles, particularly at low ionic strength, but can be further enhanced by kinetically trapping the droplet into a distinct core-shell morphology.

### Angular-independent colour from photonic pigments

**Fig. 7:**
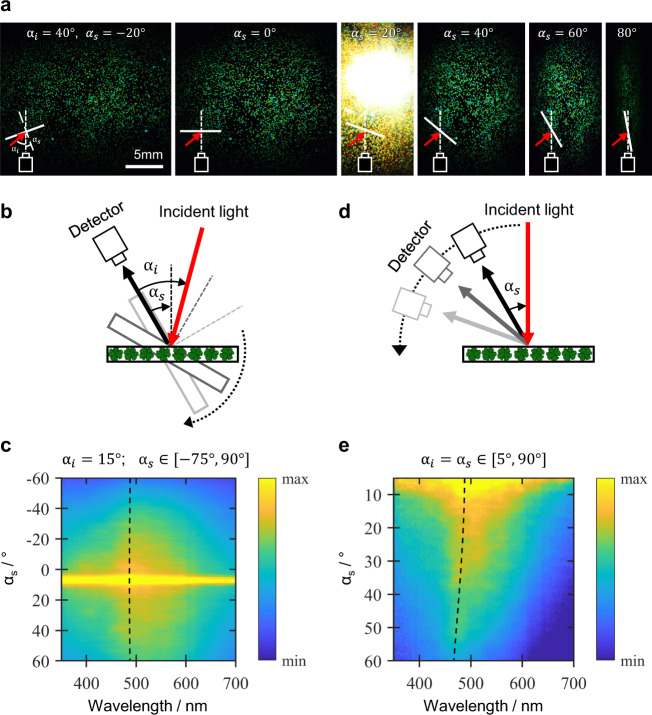
Angular dependence analysis of the reflected colour of coatings containing CNC photonic microparticles. **a** Photographs showing a consistent green appearance for a PDMS film embedded with CNC photonic pigments, upon rotating the sample with a fixed illumination and viewing angle. Specular reflection from the film interface is at ${\alpha }_{s}$ = 20° (**b**–**e**) Angular resolved optical spectroscopy of the same PDMS film recorded under two complementary sets of illumination and viewing conditions, as described in the schematics. Both conditions show a limited angular dependence for the reflection peak over this wide angular range.

To demonstrate the potential use of the CNC microparticles as photonic pigments within a paint or coating, green microparticles were embedded in a polydimethylsiloxane (PDMS) film and optically characterised under two representative observation conditions. In the first configuration, the embedded film was observed at a fixed illumination angle (${\alpha }_{i}$) with respect to the viewing direction and the sample itself was rotated at different angles (${\alpha }_{s}$) defined with respect to the viewing direction (Fig. 7a, b). Direct observations showed green reflection with no detectable colour change for the explored angles (${\alpha }_{s}$). Angular-resolved optical spectroscopy confirmed the optical response remained centred at the same wavelength (approximately 490 nm, Fig. 7c). In the second observation configuration, the sample was illuminated at normal incidence and the viewing direction was scanned, which corresponds to maintaining equal illumination (${\alpha }_{i}$) and sample $({\alpha }_{s})$ angles, such that ${\alpha }_{i}={\alpha }_{s}\in [0^\circ ,90^\circ ]$, Fig. 7d). In this case, only a limited blueshift in the peak wavelength (i.e., $\Delta \lambda \approx$ 25 nm, $\Delta \lambda /\lambda$ <5%) was detected (Fig. 7e). These two complementary configurations, performed in off-specular conditions as defined with respect to the film interface (i.e., ${\alpha }_{i}\ne 2{\alpha }_{s}$), demonstrate the non-iridescent character of the microparticles when embedded in the PDMS matrix, which sharply contrasts with the strong iridescence observed for dish-cast CNC films^12^.

The origin of the angular independence can be explained by a simple model based on ray-tracing analysis. As the macroscopic angular optical response of the buckled CNC microparticles matches surprisingly well with that of a radially aligned cholesteric sphere (Supplementary Fig. 17)^28,50^, each embedded CNC microparticle was approximated by a microsphere with a concentric multi-layer architecture (Supplementary Fig. 18). Due to Snell’s law, the light reflected from the microparticle has a critical maximum angle that allows for transmission through the PDMS-air interface (Supplementary Fig. 19). This limits the range of Bragg angles that contribute to the observed macroscopic optical response. From this model, the maximum expected spectral shift is limited to $\Delta \lambda /\lambda$ <0.5% in the first configuration and $\Delta \lambda /\lambda$ <6.5% in the second configuration (Fig. 4b, d), validating the use of these microparticles as non-iridescent photonic pigments.

## Discussion

Starting from a commercially available CNC suspension, we successfully prepared cellulose photonic pigments that can reflect colour across the full visible spectrum, overcoming the large pitch inherent to spheroidal CNC microparticles. The key to additional pitch compression was interfacial buckling, which was achieved by an appropriate initial CNC source and formulation, as well as the controlled removal of residual water by either thermal or polar solvent post-treatment. The interfacial buckling distorted the helicoidal architecture of the microparticles, giving rise to reflection of both LCP and RCP light, which is rarely observed for CNC films. Importantly, cellulose photonic pigments embedded in a matrix display angular independent structural colour, in contrast to the iridescence of drop-cast photonic films. Finally, while recent progress has been made to upscale photonic CNC film production^39^, using an emulsion-based route allows for pigments to be directly produced in a single step without the need for a substrate. Furthermore, due to the good colour tolerance with respect to the microparticle size, this approach should be transposable from microfluidics to larger scale emulsion methods, such as membrane emulsification, which would enable them to be produced via a continuous fabrication process. As such, these cellulose photonic pigments offer a sustainable, biocompatible and scalable solution to the colourant industry, where there is a demand to transition away from synthetic polymers and unrenewable minerals to those derived from natural materials.

## Methods

### Cellulose nanocrystal suspension

The cellulose nanocrystal suspension was purchased from the Process Development Center of the University of Maine (batch no. 2015-FPL-077). The as-received suspension was provided pH-neutralised (i.e., negatively charged CNCs due to –OSO_3_^−^ groups, with Na^+^ counter-ions), and was used as received, i.e., no tip sonication or thermal treatment was applied. The measured concentration of the suspension was 10.7 wt.% (A&D, MX-50 moisture analyser). Consistent with previous benchmarking studies^51^, the zeta potential and Z-average size were respectively recorded to be −43.5 ± 1 mV and 121 ± 2 nm (Malvern Zetasizer Nano ZS; [CNC] = 0.25 wt.%, [NaCl] = 5 mm, passed through a 0.8 μm cellulose acetate syringe filter). A 6.0 wt.% dilution of this commercial suspension had a conductivity of 477 ± 6 μS cm^−1^ (Mettler Toledo InLab 752-6MM) and a pH of 4.8 (Mettler Toledo InLab Micro Pro-ISM; measured in the presence of an excess of K^+^ ions^52^). Conductometric titration of an acidified CNC suspension (0.5 wt.%, 20 mL) against sodium hydroxide (0.01 m) revealed [–OSO_3_^(−)^] = 398 μmol g^−1^ and [–COO^(−)^] = 9 μmol g^−1^. The acidified suspension was prepared by ion exchange with sulfuric acid, followed by extensive dialysis against deionised water. An example TEM image of the elongated, splinter-like CNC nanoparticles is included in Supplementary Fig. 20, along with a phase diagram for the as-received suspension.

The commercial suspension was diluted with Milli-Q water and aqueous sodium chloride solution (0.1 M) to obtain suspensions with 7.0 wt.% of CNC and a [NaCl]/[CNC] ratio of 85–100 μmol g^−1^. The suspensions were then equilibrated for at least seven days to allow for phase separation (e.g., for 100 μmol g^−1^: 82% anisotropic phase, with c_iso_ ≈ 6.5 wt.% and c_aniso_ ≈ 7.1 wt.%), and the denser anisotropic phase was used as standard to prepare the emulsions. Alternative formulations were prepared following a similar procedure, as summarised in the flow chart in Fig. 6.

### Microdroplet generation

Monodisperse microdroplets were generated within a hydrophobic, etched-glass microfluidic device containing a 105 μm wide flow-focusing junction and a channel depth of 100 μm (Dolomite, #3000158). The dispersed phase was the aqueous CNC suspension and the continuous phase comprised of hexadecane (Sigma-Aldrich) with 2.0 wt.% of Span 80 surfactant (Sigma-Aldrich). To generate monodisperse water-in-oil droplets (Ø ≈ 160 μm), the aqueous and oil phases were injected into the microfluidic device via two syringe pumps (Harvard Apparatus, PHD 2000) with respective flow rates of 800 and 7200 μL h^–1^ (Nb. droplets with other sizes were achieved by tuning the flow rate ratio). To produce microparticles, 200 μL of CNC droplets were collected into a 9 cm polystyrene Petri dish filled with 15 mL of 0.5 wt.% of Span 80 in hexadecane and left to dry, with a typical drying time of 2–3 days. Notably, microdroplets dried much more slowly (i.e., over several weeks) did not show any differences in the resultant microparticle morphology or optical response.

### Solvent treatment of the CNC microparticles

The dried microparticles were washed several times with *n*-hexane (Fisher) to replace the non-volatile hexadecane and to remove residual surfactant. Residual *n*-hexane was evaporated from the microparticles under a flow of nitrogen to yield a fine, free-flowing powder. To further blueshift the CNC microparticles, they were immersed in either isopropanol (Fisher) or methanol (Fisher) for two minutes and again blown dry to evaporate any residual solvent. The red, green and blue samples presented in this work were prepared from a 7.0 wt.% CNC suspension, with the ionic strength and solvent treatment varied to fine-tune the final colour of the microparticles, as summarised in Supplementary Table 2.

### Thermal treatment of the CNC microparticles

The *n*-hexane washed microparticles were dried on a coverslip and placed in a heating chamber (Linkam Scientific, THMS600). Dry nitrogen gas was flushed through the chamber while the sample was held at a specific temperature for 60 min. The initial heating rate was set to 20 °C min^−1^. The decrease in microparticle size was observed to occur over the first 30 min of heating (Supplementary Fig. 7).

### Preparation of microparticle-embedded films

Suspending the CNC microparticles in a PDMS matrix allows for them to be well dispersed within a film geometry, while also removing interfacial scattering from the individual microparticles. PDMS and the cross-linker (Sylgard 184 elastomer kit, Dow Corning) were mixed in a 10:1 weight ratio, prior to blending with a powder of green CNC microparticles. Air bubbles were then removed from the mixture under reduced pressure until the dispersion was no longer cloudy. The mixture was poured onto a glass slide and allowed to cure at room temperature for approximately 24 h. The microparticles did not change colour during the curing process.

### Preparation of a uniformly aligned dish-cast photonic CNC film^53^

2 mL of a 50% dilution of the aforementioned stock CNC suspension (i.e., the anisotropic phase of a 7.0 wt.% CNC suspension with [NaCl]/[CNC] ratio 100 µmol g^−1^) was placed in a petri dish (Corning 430588, Ø = 35 mm, nontreated polystyrene) and inserted in the gap between a pair of NdFeB magnets (First4magnets, F390-N42, L40 × W40 × H30 mm^3^) with vertically aligned magnetisation (vertical gap = 24 mm, estimated field *µ*_0_*H* ≈ 0.6 T). The CNC suspension was left to slowly dry over four days under an upturned glass beaker (3 L) at ambient conditions and the resultant transparent film characterised by UV-vis spectrometry (Cary 4000 spectrophotometer) and cross-sectional SEM analysis (see below).

### Optical characterisation

Prior to imaging, the microparticles were dispersed in refractive index-matching oil (Cargille Series A, ${n}_{D}^{25}$ = 1.5500) to reduce broadband scattering from the particle-oil interface. Note, no swelling or colour change of the microparticles was observed when immersed in this oil. Reflection optical micrographs were collected with a customised Zeiss Axio scope A1 microscope fitted with a CMOS camera (Eye IDS, UI-3580LE-C-HQ, calibrated with a white diffuser) using a Halogen lamp (ZEISS, HAL100) as a light source in Koehler illumination. All bright-field and dark-field images were taken with a Zeiss EC Epiplan-Apochromat objective (×20, NA 0.6), with the microscope configured such that the numerical aperture (NA) in illumination was limited by the NA of the objective. The reflected light could also be filtered with a quarter wave plate and a linear polariser mounted at different orientations to distinguish between left- or right-handed circularly polarised light. To perform micro‐spectroscopy, the microscope was coupled to a spectrometer (Avantes, AvaSpec‐HS2048) using an optical fibre (Avantes, FC‐UV200‐2‐SR, 200 µm core size) in confocal configuration. The reflectance spectra were normalised in dark-field in left-circular polarisation against a white diffuser (Labsphere SRS‐99‐010) coated with the same refractive index oil (Supplementary Fig. 21). The timelapse series were recorded on an Olympus IX-71 inverted microscope, using Olympus UPlanFLN (×10, NA 0.30) and LUCPlanFLN (×40, NA 0.60) objectives with an additional 1.6x magnifying lens, and imaged with a CMOS camera (Pixelink PL-D725CU-T). Where noted, transmission images were collected through crossed polarisers with an additional full-wave retardation (i.e., tint) plate (Olympus, U-TP530) to both enhance the overall brightness and contrast of the image, and to indicate the orientation of the cholesteric domains. Photographs of vials containing microparticles dispersed in ethyl cinnamate (Sigma-Aldrich, ${n}_{D}^{20}$ = 1.558) were recorded under diffuse illumination (i.e., fluorescent ceiling light) with a Samsung Galaxy S9+ smartphone.

### Angular-resolved optical spectroscopy

A laboratory-built goniometer was used to analyse the angular response of the CNC microparticles. A white light source (Schott KL1500 set to 3300 K) was used to illuminate as a collimated incident beam with a spot size of approximately 6 mm and at a fixed angle of 0°. The detector was mounted on an arm attached to a motorised rotation stage and coupled the light into an optical fibre (1000 μm core) connected to a spectrometer (Avantes AvaSpec-HS2048XL). The sample was mounted on a rotation stage at the centre of the goniometer. The recorded light intensity was normalised with respect to the white Lambertian diffuser in air, while the exposure time was adjusted using an automatised high-dynamic-range (HDR) method^53^. For the microparticle-embedded film, the first measurement was collected while the sample was rotated between −90° and +75° with respect to the illumination source (setting the origin of the angles), with the detector fixed at −15° with respect to the illumination source. In the framework of the observer, this corresponds to an angular scan of the sample orientation ${\alpha }_{s}$ = [+90, −75] (as defined by the normal of the film surface, Fig. 7b) at fixed illumination angle ${\alpha }_{i}$ = +15﻿°. The second measurement was collected with the sample fixed at 0° and by scanning the detector between 0° and +90°. In the framework of the observer, this corresponds to an angular scan of ${\alpha }_{i}$ = ${\alpha }_{s}$= [0,90] (Fig. 7d). For the microparticle dispersion, the vial was fixed, and the detector was scanned between +45° and +185° (Supplementary Fig. 18a). Angular-resolved photography was taken with a digital camera at a fixed position (Nikon, D3200, with a 68 mm macro extension tube), with either the sample or light source rotated.

### Thermogravimetric Analysis (TGA)

CNC microparticles were evaluated using a TGA/DSC 2 instrument (Mettler Toledo) using an aluminium pan and with the following parameters: a nitrogen flow of 100 mL min^−1^, heating rate of 1 °C min^−1^, temperature range of 30–300 °C, sample mass of 5–10 mg.

### Scanning electron microscopy (SEM)

Micrographs were collected with a Mira3 system (Tescan) operated at 5 kV and a working distance of 6–7 mm. The samples were mounted on aluminium stubs using conductive carbon tape and coated with a 10 nm thick layer of platinum with a sputter coater (Quorum, Q150T ES). To image the exterior of the microparticles by SEM, the microparticles were dispersed in *n*-hexane and the suspension was drop cast onto a glass coverslip followed by drying with nitrogen. To image the interior of microparticles, they were cryo-fractured using the following protocol: (i) the microparticles in *n*-hexane were drop cast on a coverslip, (ii) embedded in cellulose butyrate acetate resin (10 wt.% cellulose butyrate acetate dissolved in ethyl acetate), (iii) transferred into a dry nitrogen atmosphere, (iv) frozen in liquid nitrogen for 5 min, (v) mechanically crushed.

### Transmission electron microscopy (TEM)

Micrographs were captured using a Talos F200X G2 microscope (Thermo Scientific, FEI) TEM operating at 200 kV and a CCD camera. Samples were prepared as follows: (i) The CNC suspension was diluted in ultrapure water in two successive steps, first to 0.1 wt.% then to 0.005 wt.%. (ii) A TEM grid (Agar Scientific S160-3 Carbon film 300 mesh Cu) was plasma treated, followed by deposition of a drop of the 0.005 wt.% CNC suspension. This was left to sit on the grid for 90 s before gently removing all residual liquid with filter paper (Whatman). (iii) The CNCs were stained with uranyl acetate solution for 60 s, with excess liquid again removed by blotting.

## Supplementary information

Supplementary Information

Description of Additional Supplementary Files

Supplementary Movie 1

## Data Availability

All raw data relating to this publication is freely accessible from the University of Cambridge data repository (10.17863/CAM.72840).
