# Supplementary material for: Cellulose photonic pigments
Source: Nat Commun. 2022 Jun 13;13:3378. doi: 10.1038/s41467-022-31079-9 (PMC9192732; doi:10.1038/s41467-022-31079-9)
Supplement: Supplementary file 1 — Supplementary Information [file 41467_2022_31079_MOESM1_ESM.pdf]

Supplementary Information

Cellulose Photonic Pigments

Dr Richard M. Parker[1 †], Dr Tianheng H. Zhao[1 †], Dr Bruno Frka-Petesic[1], and Prof Silvia Vignolini[1 *]

[1] Yusuf Hamied Department of Chemistry, University of Cambridge, Lensfield Road, Cambridge, CB2 1EW, United Kingdom.

[†] Richard M. Parker and Tianheng H. Zhao contributed equally to this work.

* Correspondence to Prof Silvia Vignolini (sv319@cam.ac.uk).

Supplementary Discussion

Discussion 1: Analysis of TGA data

During the heating of both untreated and methanol-treated samples, the CNC microparticles experienced water loss, desulfation and eventually degradation of the cellulose, which all contribute to the mass loss observed in the TGA curves (Nb: TGA curves of the remaining mass fractions are subsequently referred to as $\text{TGA}_{\text{untreated}}(T)$ and $\text{TGA}_{\text{methanol}}(T)$). The methanol-treated sample experienced $w_{\text{MeOH},2} = \text{TGA}_{\text{methanol}}(50) - \text{TGA}_{\text{methanol}}(200) = 4\%$ of mass loss prior to 200 °C. This mass loss is likely to be due to residual methanol that was not fully removed after the solvent treatment. The expected volume decrease (with respect to 50 °C, which was taken as the reference) is thus expected to be $\frac{V_{200°\text{C},2}}{V_{50°\text{C},2}} = \frac{\rho_{\text{CNC}}^{-1} w_{\text{CNC},2}}{\rho_{\text{CNC}}^{-1} w_{\text{CNC},2} + \rho_{\text{MeOH}}^{-1} w_{\text{MeOH},2}}$ and $\frac{d_{200°\text{C},2}}{d_{50°\text{C},2}} = \left(\frac{V_{200°\text{C},2}}{V_{50°\text{C},2}}\right)^{1/3} \approx 0.9734$ using ρ_{CNC} =1.600 g cm^{-3} and ρ_{MeOH} =0.792 g cm^{-3} (and $w_{\text{CNC},2}$ = 1- $w_{\text{MeOH},2}$). From 200 °C to 250 °C, a further mass loss $w_{\text{sulf},2} = \text{TGA}_{\text{methanol}}(200) - \text{TGA}_{\text{methanol}}(250)$= 6% is attributed to the desulfation of the CNCs, so that $w_{\text{desulfCNC},2}$=$\text{TGA}_{\text{methanol}}(250) = 90\%$. If we assume this desulfation to be independent of the solvent, we can consider the relative mass loss due to desulfation in methanol-treated particles is proportional to the amount of remaining cellulose in the untreated particles, so $\frac{w_{\text{CNC},2}}{w_{\text{desulfCNC},2}} = \frac{w_{\text{CNC},1}}{w_{\text{desulfCNC},1}}$, where the subscripts 1 and 2 refer, respectively, to untreated and the methanol treated samples. The desulfated CNC mass fraction in the untreated sample is taken as $w_{\text{desulfCNC},1} = \text{TGA}_{\text{untreated}}(250)$, from which we get $w_{\text{CNC},1} = 69.65\%$ and $w_{\text{water},1} = 1 - w_{\text{CNC},1}$. The corresponding initial volume before water evaporation is estimated as $\frac{V_{50°\text{C},1}}{V_{200°\text{C},1}} = \frac{\rho_{\text{CNC}}^{-1} w_{\text{CNC},1} + \rho_{\text{water}}^{-1} w_{\text{water},1}}{\rho_{\text{CNC}}^{-1} w_{\text{CNC},1}} \approx 1.697$ and the diameter ratio as $\frac{d_{50°\text{C},1}}{d_{200°\text{C},1}} = \left(\frac{V_{50°\text{C},1}}{V_{200°\text{C},1}}\right)^{1/3} = 1.1928$. The diameter ratio expected between the untreated and treated particles is then estimated as $\frac{d_{50°\text{C},2}}{d_{50°\text{C},1}} \approx \frac{1}{1.1928} * \frac{1}{0.9734} \approx 0.8612$, i.e., a decrease of 13.9%.

Another relevant observation is the difference at which the two TGA curves seem to plateau at high temperature, indicating the mass of solid carbon atoms (C) remaining from the pyrolysis of CNCs: $w_{\text{C},1} = \text{TGA}_{\text{untreated}}(300) \approx 17.26\%$ and $w_{\text{C},2} = \text{TGA}_{\text{methanol}}(300) \approx 39.73\%$. After rescaling by their respective proportion of CNC content in each sample, we obtain the ratios $r_{(\text{C/CNC}),1} = \frac{w_{\text{C},1}}{w_{\text{CNC},1}} \approx 23.8\%$ and $r_{(\text{C/CNC}),2} = w_{\text{C},2}/w_{\text{CNC},2} \approx 39.7\%$. This has to be compared to the expected mass ratio of six carbon atoms in cellulose ($(C_6H_{10}O_5)_n$), which gives $r_{(\text{C/CNC}),\text{max}} = 44.45\%$ and represents a theoretical limit if all C atoms are retained in the solid residue, namely $\frac{r_{(\text{C/CNC}),1}}{r_{(\text{C/CNC}),\text{max}}} = 53.5\%$ and $\frac{r_{(\text{C/CNC}),2}}{r_{(\text{C/CNC}),\text{max}}} = 89.4\%$. According to D.-Y. Kim *et al.*,[1] the presence of adsorbed water allows for the reaction of oxygen atoms (present in water) to react with carbon and release CO and CO_2 gas, thus decreasing the ratio of the remaining solid carbon residue. This rescaling makes the excess water in the initial untreated particles more apparent, but also indicates that at 300 °C, methanol treatment significantly prevents mass loss *via* pyrolysis, approaching the theoretical maximum (green dashed line in Supplementary Figure 10). This confirms the role of methanol as a dehydrant, but also indicates that, since some CO and CO_2 has formed (both $r_{(\text{C/CNC}),1}$ and $r_{(\text{C/CNC}),2}$ < 1), the above estimations assuming complete dehydration at 250 °C are only indicative.

Discussion 2: Scaling analysis of pitch compression in the presence of buckling

In the following discussion a qualitative description of the pitch scaling behaviour is proposed based on a stepwise introduction of shrinkage, local anisotropic distortion, and global buckling transformations, which are treated sequentially for the sake of clarity, but expected to occur in parallel. This pitch scaling is relevant only after the kinetic arrest of the suspension has occurred.

Qualitatively, the pitch shrinkage upon water loss that is expected in the absence of buckling for a kinetically arrested Frank-Pryce structure can be estimated from a simple scaling law. Let us define an initial spherical droplet of radius R_0, surface $S_0 = 4\pi R_0^2$, volume $V_0 = 4\pi R_0^3/3$ and pitch $p_0 = R_0/N$, where N is a constant. The cross-sectional area of the droplet is $\Sigma_0 = \pi R_0^2$ and its perimeter is $\mathcal{P}_0 = 2\pi R_0$. The volume fraction of CNCs in the droplet is defined as ϕ_0 and the solid angle of the surface viewed from the centre is $\Omega_0 = S_0/R_0^2 = 4\pi$. This initial state corresponds to the droplet at the onset of the kinetic arrest.

After a radial contraction α $(0 < \alpha \leq 1)$ in the absence of buckling, these quantities are expected to scale as $R_1 = \alpha\, R_0$, $S_1 = \alpha^2\, S_0$, $V_1 = \alpha^3\, V_0$, $p_1 = \alpha\, p_0$, $\Sigma_1 = \alpha^2 \pi R_0^2$ and $\mathcal{P}_1 = 2\pi\, \alpha R_0$, respectively. The volume fraction of CNCs in the droplet is given by $\phi_1 = \alpha^{-3}\phi_0$ and the solid angle of the surface viewed from the centre is unchanged $\Omega_1 = S_1/R_1^2 = 4\pi$.

Let us introduce a distortion term β $(\alpha \leq \beta \leq 1)$ to account for a local asymmetry of the contraction of the cholesteric structure, whereby the compression along the helical axis is facilitated. The aforementioned quantities in this distorted particle can, in first approximation, be expected to scale as $R_2 = \beta^2\, R_1 = \alpha\beta^2 R_0$, $S_2 = \beta^{-2} S_1 = \alpha^2\beta^{-2}\, S_0$, $V_2 = V_1 = \alpha^3\, V_0$, $p_2 = \beta^2\, p_1 = \alpha\beta^2\, p_0$, $\Sigma_2 = \alpha^2 \pi R_0^2$ and $\mathcal{P}_2 = 2\pi\, \alpha\beta^{-1} R_0$, respectively. The volume fraction remains unaffected, i.e., equal to $\phi_2 = \phi_1 = \alpha^{-3}\phi_0$, resulting in the relationship $\alpha = (\phi_2/\phi_0)^{-1/3}$ that will prove useful later. Note, the resulting solid angle of this larger surface, when viewed from the centre, is $\Omega_2 = S_2/R_2^2 = 4\pi\beta^{-6} \geq 4\pi$, which causes an expanded solid angle that is inconsistent to maintain an unbuckled spherical shape if $\beta < 1$. The distortion term β intervenes thus both in the pitch p_2 and in the perimeter $\mathcal{P}_2$. We can estimate β from cross-sectional SEM analysis of a buckled microparticle, by measuring Σ_2 and $\mathcal{P}_2$ and deducing from it $\beta^{-2} = \mathcal{P}_2^2/(4\pi\Sigma_2)$, as reported in Supplementary Figure 11. The inverse quantity, $Q = 4\pi\Sigma_2/\mathcal{P}_2^2$, is also known as the isoperimetric quotient, such that we get $Q = \beta^2 = p_2/p_1$. The pitch p_1 (a so-called 'Q-corrected pitch' that represents the pitch in the absence of buckling) can thus be experimentally estimated from the measured p_2 of the microparticles and from their corresponding Q using $p_1 = p_2/Q$.

While this modelling allows a prediction of the pitch p_2 for a given distortion term Q, it poses no constraints on the possible relationship between Q and ϕ_2, or in other words, between α and β. Various scenarios are possible for the pitch function $p_2(\phi)$, then defined as the pitch at a given volume fraction ϕ in the buckled droplet or particle rather than only in the final solid particle,. Several cases are worth discussing and are also illustrated in Supplementary Figure 22:

- The case $\beta = \alpha$ leads to a power law $p_2(\phi) = \alpha\beta^2\, p_0 = \alpha^3\, p_0 = p_0(\phi/\phi_0)^{-1}$ corresponding to vertical domains in a film geometry. This scaling is more complex for misaligned domains, which are less compressed resulting in structures with larger pitch values.[2,3] This is the reason why the dish-cast film prepared in this work was left to self-assemble under a vertical magnetic field, which favours vertical aligned domains.
- The case $\beta = 1$ leads to the unbuckled scenario $p_2(\phi) = \alpha\, p_0 = p_0(\phi/\phi_0)^{-1/3}$.
- The simple power law case, whereby buckling and kinetic arrest both occur at the same time and where the buckling term obeys a simple power law $\beta = \alpha^d$, is inconsistent with the pitch values observed in the hexane- and methanol-treated particles but agrees within the error bars with the

β values deduced from the isoperimetric quotient Q for both treatments. If such a power law is forced through (ϕ_0, p_0) and (ϕ_2, p_2) of the blue particles, we obtain $\beta = \alpha^{0.55}$ and $p_2(\phi) = \alpha\beta^2\, p_0 = p_0(\alpha/\alpha_3)^{2.4} = p_3(\phi/\phi_3)^{-0.7}$.

- The delayed buckling simple power law case corresponds to:
  - a first stage without buckling at $\beta = 1$ for the values $\phi_0 < \phi < \phi_3 \approx 0.141$ (i.e., $1 > \alpha > \alpha_3 \approx 0.768$), leading to $p_2(\phi < \phi_3) = \alpha\, p_0$ and the pitch at the onset of buckling $p_3 = \alpha_3\, p_0$.
  - followed by a buckling regime at $\phi > \phi_3 \approx 0.141$ (i.e., $\alpha < \alpha_3 \approx 0.768$) following a simple power law, where $\beta = (\alpha/\alpha_3)^{0.794} = 1.233\, \alpha^{0.794}$. This leads to $p_2(\phi > \phi_3) = \alpha\beta^2\, p_0 = p_3(\alpha/\alpha_3)^{2.588} = p_3(\phi/\phi_3)^{-0.863}$, matching the experimentally derived pitch power law between the hexane- and the methanol-treated microparticles.

  These allow for an estimation of a lower and an upper bound value for the buckling onset to $\phi_0 < \phi_{\text{Buck}} < \phi_3$ in the general case since a steeper behaviour than $\sim p_3(\phi/\phi_3)^{-0.863}$ for the pitch beyond ϕ_3 is unlikely.

- The progressive buckling case is an *ad hoc* model describing a possible scenario, whereby both kinetic arrest and buckling onset occur at the same time, yet where buckling develops progressively as the contraction evolves to more extreme values. The model proposed here is just one among many possible and was constructed with $\beta \approx \alpha^{0.853-0.718\alpha}$ and $p_2(\phi) = \alpha\beta^2\, p_0 \approx p_0\alpha^{2.707-1.437\alpha} = p_0(\phi/\phi_0)^{0.479(\phi_0/\phi)^{1/3}-0.902}$. While this model is purely phenomenological and may appear mathematically cumbersome, it illustrates well the possibility of more complex behaviours, whereby the buckling magnitude is initially weak, accounting for the relative compressibility of the soft cholesteric shell and the cholesteric domains perpendicular to their helical axes, and at a later stage gets much more pronounced, as the CNCs struggle to pack efficiently in their alignment direction, compared to the perpendicular direction.

Discussion 3: Role of the CNC formulation on the microparticle morphology

Investigating the effect of CNC formulation on the confined self-assembly pathway

To understand how the initial formulation of the CNC suspension impacts the formation of a Frank-Pryce cholesteric structure and its evolution into a buckled microparticle, three variations were prepared (see Figure 6 of the main article), with their self-assembly pathways also monitored over time and compared to the standard suspension (Supplementary Figures 12-15). From this, a proposal of the self-assembly route for each formulation variation was derived, as depicted schematically in Supplementary Figure 16.

Firstly, it was found that a simple two-fold dilution of the standard CNC suspension yielded microparticles with a much larger initial variation in colour (Figure 6c). However, upon treatment with methanol, all of the microparticles shifted to blue wavelengths. This dilution likely favoured a greater concentration gradient upon drying within the initially isotropic droplet, promoting the formation of a kinetically arrested Frank-Pryce shell, surrounding an isotropic core (Supplementary Figure 13). Upon further drying, the distinct core region continues to concentrate until it becomes anisotropic, but does not adopt radial alignment, likely due to being disfavoured by the small volume and distortion of the spherical geometry upon initial buckling. This gives rise to various shapes and water content, associated with different abilities to resist compression against centripetal capillary forces. The significant uniaxial (i.e., radial) collapse of the shell during the initial buckling process, when the liquid core is still compliant, is probably at the origin of the resulting intra- and inter-particle diversity in colour. In contrast, the more uniform blue-green colour observed after methanol treatment can then be explained by the overpowering dehydration effect that occurs regardless of the remaining water content, leading eventually to a more comparable pitch compression for all particles and orientations

upon reswelling and subsequent collapse. These results suggest that the existence of an asymmetry of the mechanical properties between a softer core and a more rigid shell favours buckling, but that a large difference is not desirable.

Secondly, microparticles produced from the corresponding isotropic phase of the standard phase-separated CNC suspension were much more spherical, with only fine surface buckling observable under SEM (Figure 6a). It was found that such microparticles display only very weak structural colour, with significant broadband scattering. Furthermore, while methanol treatment again resulted in a contraction in diameter and a blueshift, it did not enhance the intensity. These observations could be explained by the time-lapse series, which revealed that while tactoids do form upon drying the initially isotropic droplet, they do not have sufficient time to merge and organise, resulting in the formation of only a thin Frank-Pryce shell around a mostly polydomain droplet (Supplementary Figure 14). This arrangement explains the presence of only fine surface buckling and supports the idea of a thin rigid shell (with smaller pitch, where any structural colour originates) surrounding a polydomain core that did not buckle (with larger pitch, which simply scatters visible light).

Thirdly, microparticles produced from a CNC suspension formulated without any additional salt appeared colourless, which is attributed to the particles reflecting at near infrared wavelengths (Figure 6d). Such a redshift is expected upon reducing the ionic strength of the initial CNC suspension, due to the greater double layer repulsion between the CNC nanorods that typically screen the chiral interactions between CNCs, resulting in an increased pitch. Upon methanol treatment, the microparticles were observed to collapse and blueshift, but this initial offset remained, resulting in cyan rather than blue colouration. The time-lapse series showed that while the anisotropic droplet can relax and self-organise to a degree, the much higher viscosity of this suspension inhibits its ability to fully develop into a Frank-Pryce arrangement, prior to becoming trapped presumably as a Wigner glass (Supplementary Figure 15). As such, this disordered Frank-Pryce has consequently much weaker reflectivity than for the standard case (Figure 6b), confirming that long-range order is necessary to maximise the optical response. In the absence of added ions, CNCs are expected to repel each other more strongly, causing a more even distribution of the CNCs across the droplet. Unlike the previous two cases, the presence of comparable structural colour supports the idea that a strong mechanical asymmetry between core and shell is not essential and that the anisotropic compressibility of the cholesteric phase parallel vs perpendicular to the helical axis (i.e., radial vs orthoradial for a Frank-Pryce structure) could be sufficient to make the desired pitch decrease upon drying.

Comparison with microparticles prepared from a laboratory-made CNC suspension

In light of this new understanding of the role of buckling and residual water on the pitch of CNC microparticles, it is interesting to compare these observations with our previous article,[4] cited as reference 20 in the main article, where the microparticles were significantly less buckled and the measured pitch was in the micron range. Among the important differences to note, we highlight the following:

- The previous CNC suspension was laboratory-made from cotton, it was tip-sonicated, then heat-treated and no extra electrolytes were added to the formulation. The produced microparticles were not exposed to a desiccating post-treatment such as solvent or heat.
- The CNC suspension in the present work is commercially made from wood pulp and has never been sonicated. It was diluted to 7 wt.% with added NaCl to form a biphasic suspension, from which the lower anisotropic phase was used to make the emulsion. The colour of the resultant microparticles was tuned with either polar solvent or thermal post-treatments, to achieve additional desiccation.

As the two protocols differ in several aspects, identifying the key factors leading to pitch reduction and thus structural colour in the visible regime is not straightforward. For example, tip sonication is known to increase the pitch in suspension and redshift the photonic response in dried films, while heat treatment is known to cause desulfation, resulting in reduced surface charge and release of electrolytes, both leading to a blueshift. However, the impact of such treatments on the onset of kinetic arrest, is still not well understood. This is particularly important in the context of emulsified droplets where kinetic arrest leads to both significant changes in the scaling power law for pitch contraction and potentially the onset and extent of buckling.

For this reason, we also explored the effect of added electrolytes and solvent desiccation using the suspension from our previous work:[4]

(i) To understand how much the pitch can be reduced by the methods presented here and if it can lead to visible colouration, the ionic strength of the previously reported CNC suspension[4] was increased from 74 μmol g^{-1} to 115 μmol g^{-1} of sulfuric acid per CNC. This had the result of reducing the pitch within the microparticles from 1200-1400 nm (i.e. 1.2-1.4 μm) to 700-800 nm. However, further increasing the acid concentration to 212 μmol g^{-1}, was found to result in spheroidal particles (i.e. negligible buckling) that did not show significant self-organisation of the cholesteric phase during the drying process (data not shown). As such, while suspension optimisation can be used to significantly reduce the pitch, this alone is insufficient to reach visible wavelengths. Note for simplicity, the electrolyte added to the heat-treated suspension was sulfuric acid, but salt (such as NaCl) was seen to act similarly.

(ii) The effect of a solvent post-treatment was also explored. Microparticles prepared from the optimised formulation (i.e. 115 μmol g^{-1}) were washed with methanol and subsequently dried. This resulted in a reduced size with increased buckling across the surface. Moreover, very localised patches of red colour could be observed on some microparticles under the microscope. Cross-sectional SEM confirmed that the pitch had decreased to around 400-500 nm in the limbs, corresponding to reflection predominantly at infrared wavelengths (data not shown).

As such, while the approaches reported in this article can significantly reduce the pitch, it was not possible with the previous CNC suspension to produce visibly coloured microparticles.

Beyond the role of isolating the various directly accessible parameters, such as those exemplified above, the more fundamental parameters to consider are most likely the pitch before kinetic arrest and the volume fraction of CNCs at the onsets of both kinetic arrest and then buckling. These three quantities could, by themselves, be sufficient to describe the evolution of the pitch upon volume contraction until the formation of the final microparticles. While the pitch before kinetic arrest determines the overall starting position in the pitch *vs* volume fraction diagram (see Figure 5), the kinetic arrest transition defines where the pitch contraction suddenly decreases to $p \sim \Phi^{-1/3}$. As such, an early buckling transition, i.e., before most of the water is lost, is then essential to not only to restore a steeper pitch evolution, comparable to the $p \sim \Phi^{-1}$ we find in vertically aligned domains in dried films, but to allow sufficient drying time to exploit this pitch contraction and reach pitch values consistent with the visible range. Such parameters are likely related to the morphology of the CNC nanoparticles themselves and as such are likely related to the cellulose source and the extraction conditions used to yield the initial CNC suspension.

Supplementary Tables

Sample	N (sample #)	μ / µm (mean)	σ / µm (std dev)	err_{μ} / µm (SEM)	$\Delta\mu = \frac{(\mu_2 - \mu_1)}{\mu_1}$	$err_{\Delta\mu}$ (SEM)
1 - no methanol	26	88.5	4.12	± 0.81	-12.1%	±1.1%
2 - methanol	20	77.9	2.26	± 0.50		

Supplementary Table 1. Statistical analysis of the diameter Ø of the CNC microparticles from SEM analysis (i = 1: no methanol), and its variation where a methanol treatment was previously applied (i = 2: methanol). Here μ_i and σ_i correspond to the mean and standard deviation of Ø in the sample i. The *standard error of the mean of* Ø (i.e., error on μ_i) is estimated as $err_{\mu_i} = \sigma_i / N_i$, where N_i is the number of measured particles. The variation of the mean diameter is calculated as $\Delta\mu = (\mu_2 - \mu_1)/\mu_1$, while $err_{\Delta\mu}$ quantifies its *standard error*, obtained by error propagation of their respective err_{μ_i} terms:

$$\frac{err_{\Delta\mu}}{\Delta\mu} = \sqrt{\frac{err_{\mu_2}^2 - err_{\mu_1}^2}{(\mu_2 - \mu_1)^2} + \frac{err_{\mu_1}^2}{\mu_1^2}}.$$

Pigment colour	CNC / wt.%	[NaCl]/[CNC] / µmol g^{-1}	Solvent post-treatment
red	7.0	100	none
green	7.0	85	isopropanol
blue	7.0	100	methanol

Supplementary Table 2. Suspension formulation and microparticle treatment conditions to produce the red, green, and blue photonic CNC pigments reported in Figure 1 of the article. Note that while solvent treatment can result in large, discrete changes in the colour of the microparticles, the ionic strength of the initial formulation can also be used to fine-tune the final optical appearance, as was employed here to achieve a visibly green dispersion after isopropanol treatment (85 µmol g^{-1}), rather than cyan in appearance (100 µmol g^{-1}). The solvent post-treatment was not limited to those in this table, but validated for a wide range of polar solvents, including: methanol, ethanol, isopropanol, *t*-butanol acetone, acetonitrile etc.

Supplementary Figures

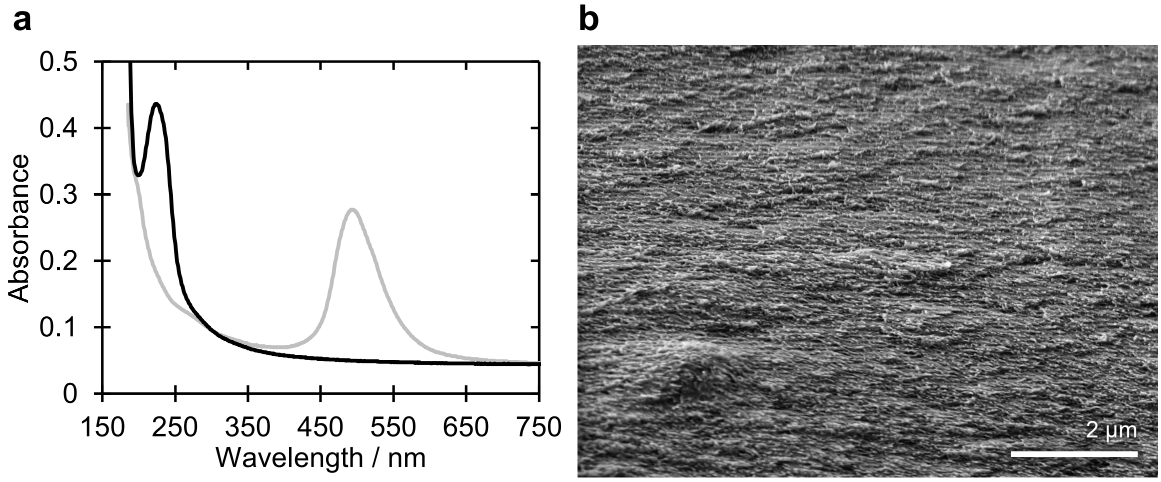

Supplementary Figure 1. Characterisation of a CNC film cast in a vertical magnetic field ($\mu_0 H \approx 0.6$ T) from a 50% dilution of the stock CNC suspension, i.e., the anisotropic phase of a commercial cellulose nanocrystal suspension (Univ. Maine, 7.0 wt.%) with [NaCl]/[CNC] ratio of 100 µmol g^{-1}. **(a)** The UV-vis spectrum of the resultant film (*black line*) shows a clear peak at $\lambda_{\max}$ = 224 nm that corresponds to a photonic response in the UVC region, and a strong absorption at <200 nm from cellulose itself, as validated against a second CNC film with visible colouration ($\lambda_{\max}$ = 493 nm, *grey line*). **(b)** The corresponding cross-sectional scanning electron microscopy (SEM) image of the UV film revealed a well-ordered helicoidal architecture with an mean pitch of 141 ± 9 nm (N = 30, uncertainty corresponds to standard deviation).

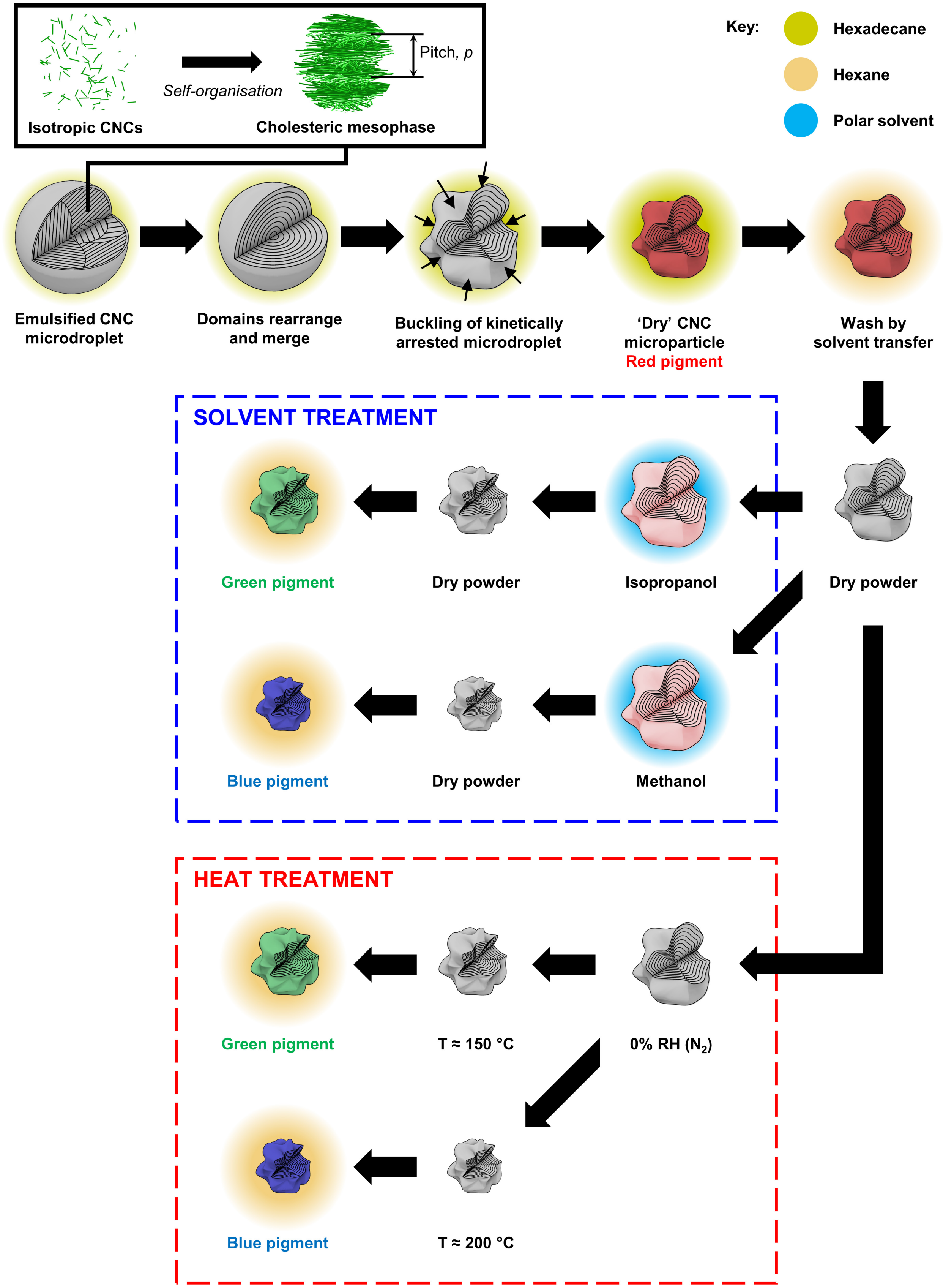

Supplementary Figure 2. An expanded schematic detailing the controlled thermal and solvent post-treatment steps to produce a full spectrum of photonic CNC pigments. The colour of the microparticle represents the typical colour reflected from the photonic nanostructure.

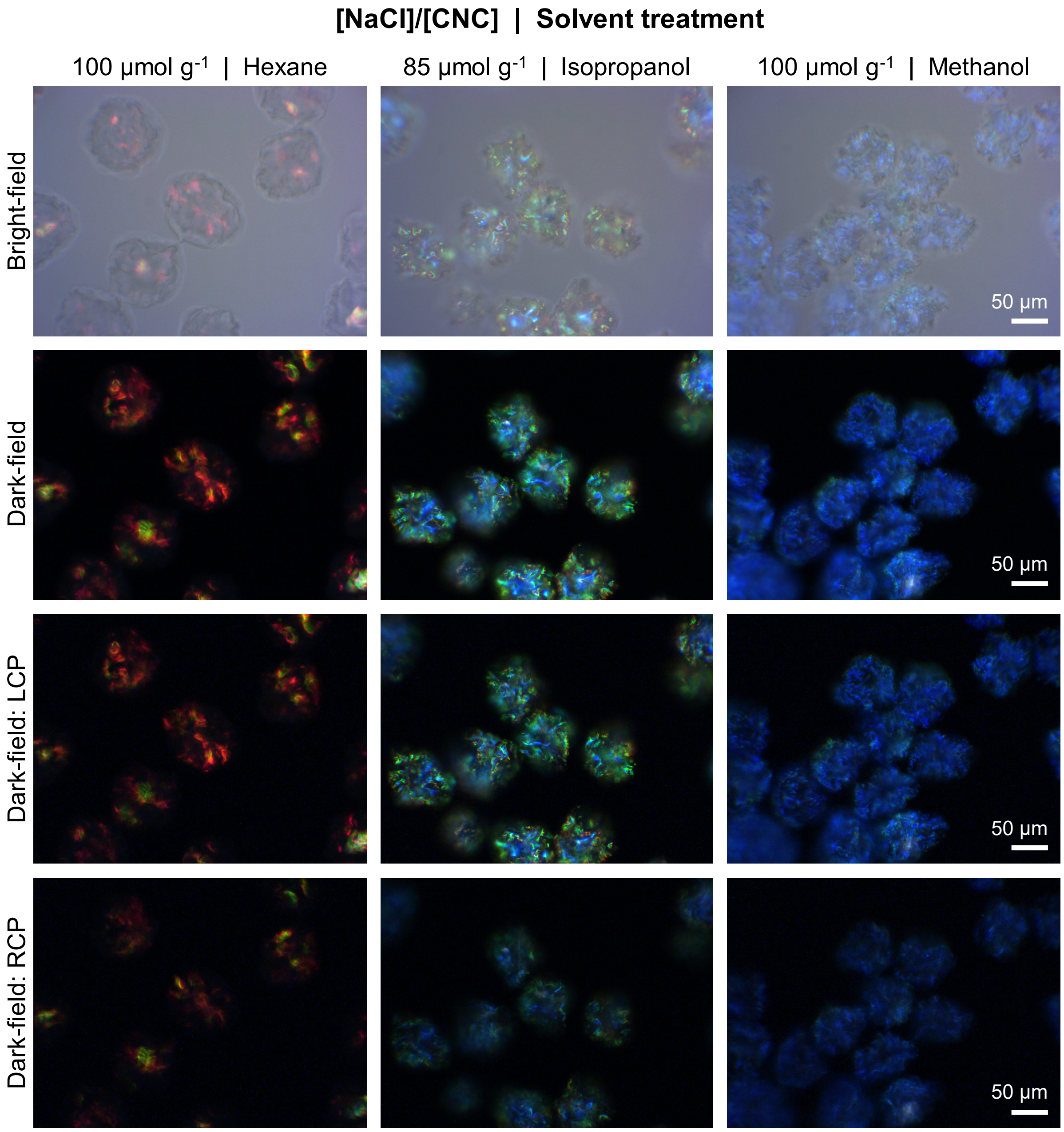

Supplementary Figure 3. Bright-field (BF) and dark-field (DF) optical microscope images of red, green, and blue microparticles (as specified in Supplementary Table 2) immersed in refractive index-matching oil (*n* = 1.55). The reflected colour is less vivid in the bright-field images due to the buckling-induced misalignment of the CNC domains that do not reflect in the specular direction of illumination, combined with the dominant specular reflection from the glass substrate that is inherent to imaging from below using an inverted microscope. The microparticles were also imaged through left-handed circularly polarisation (LCP) and right-handed circularly polarisation (RCP) filters, showing predominant reflection in the left channel.

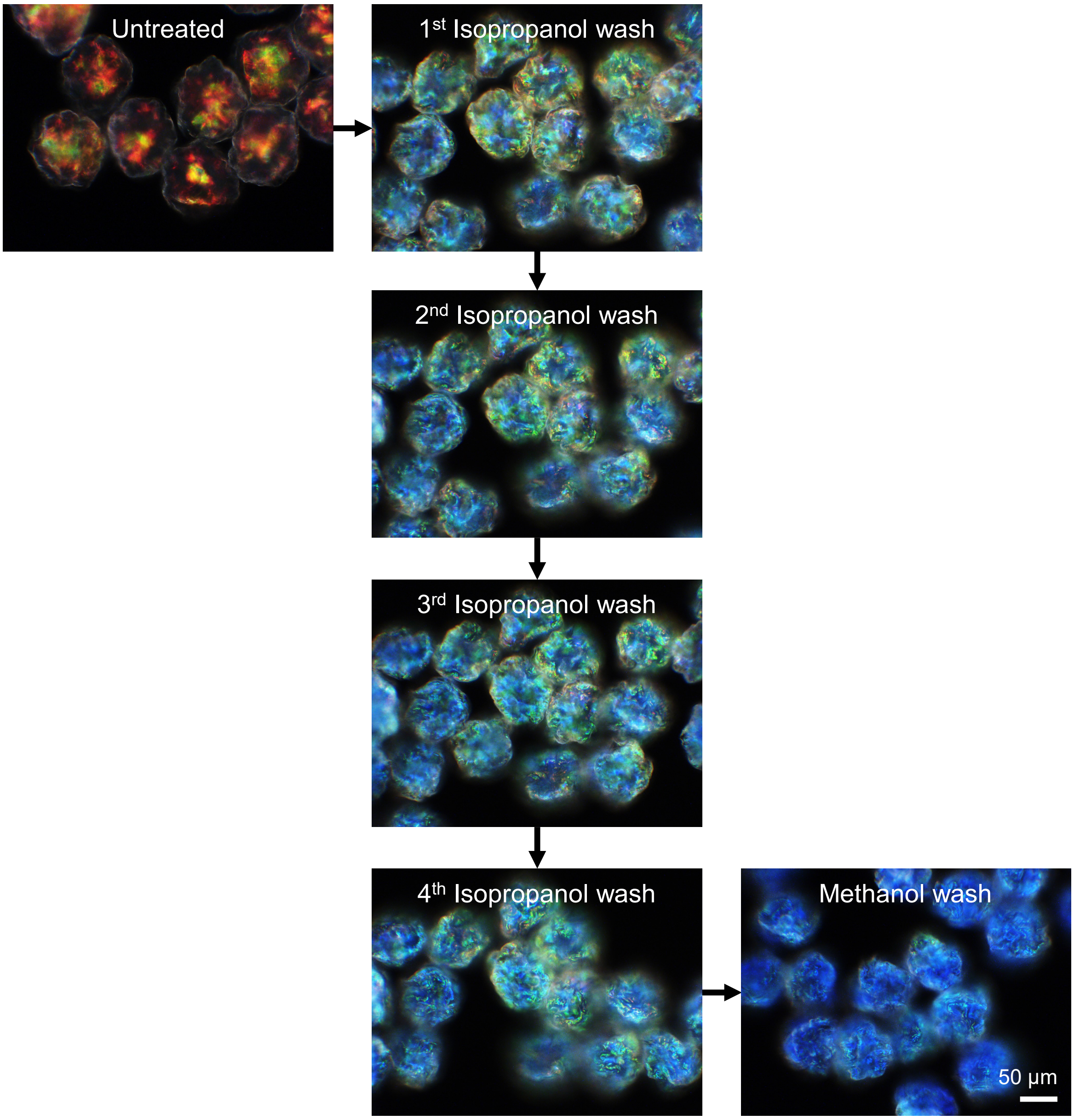

Supplementary Figure 4. Optical micrographs showing the change in the optical appearance of CNC microparticles upon successive treatments with isopropanol, and after a final treatment with methanol, a more polar solvent. After each solvent treatment, the sample was dried and immersed in non-polar hexane to remove surface scattering during imaging, as exemplified in Figure 2a-e of the article. After the first wash, additional treatments with isopropanol did not induce any significant changes to the reflected colour. However, treatment with methanol could still induce a further blueshift.

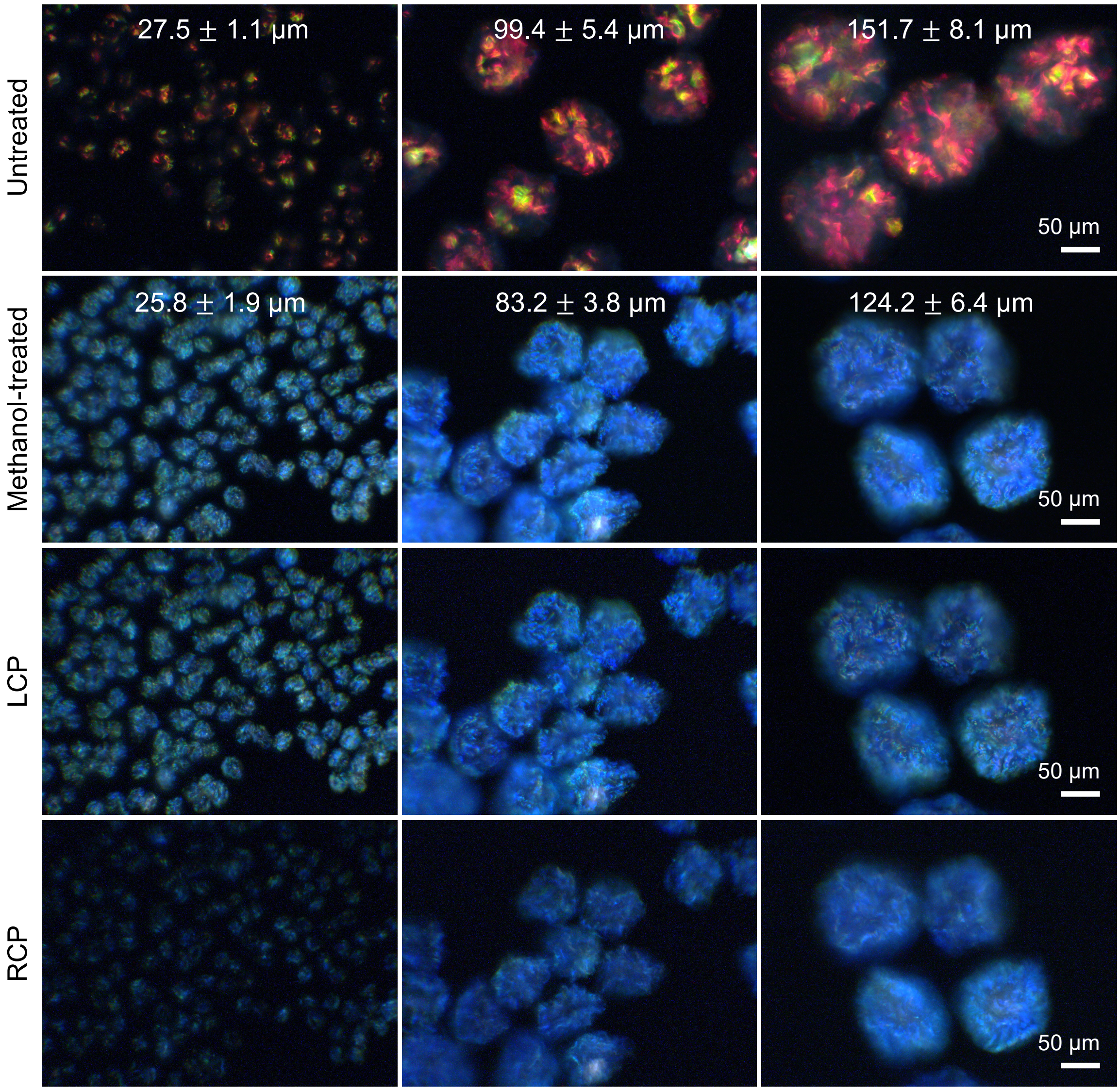

Supplementary Figure 5. Dark-field microscope images of three sizes of CNC microparticles recorded prior to, and after methanol treatment. Imaging the latter through left-handed circularly polarisation (LCP) and right-handed circularly polarisation (RCP) filters only permits light with the same polarisation to be captured, allowing the ordering of the internal architecture to be assessed.

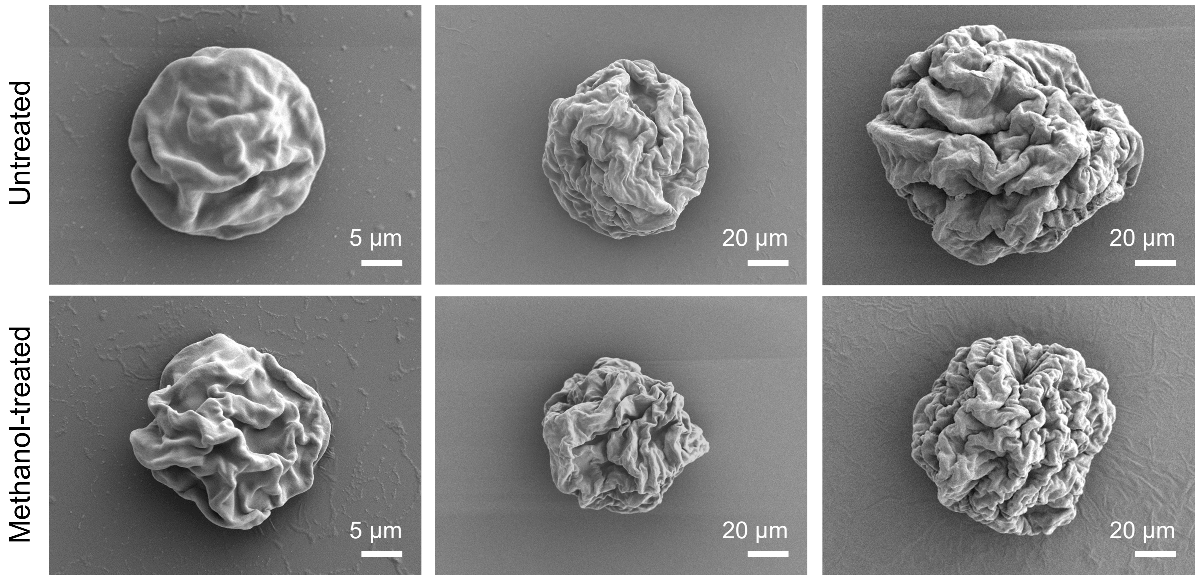

Supplementary Figure 6. Scanning electron microscope images of the exterior of a representative CNC microparticle of each of the three size regimes reported in Supplementary Figure 5, prior to (*top*) and after post-treatment with methanol (*bottom*). A decrease in diameter with a corresponding increase in surface buckling is apparent in all three cases.

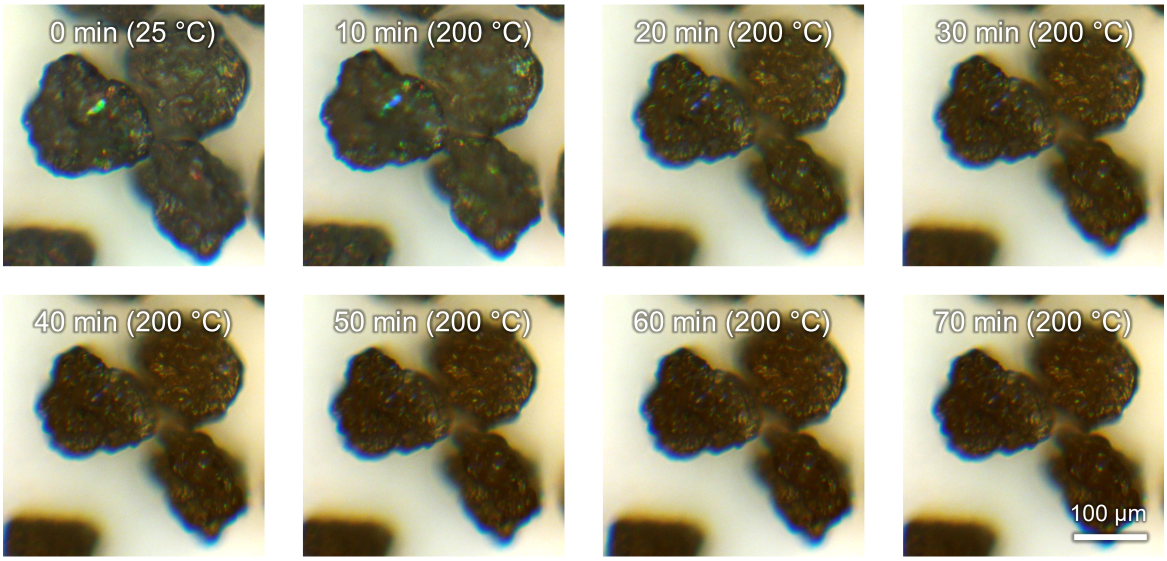

Supplementary Figure 7. Time-lapse reflectance microscope image series showing the decrease in the size of the CNC microparticle during heat treatment at 200 °C for one hour, with most of the shrinkage occurring during the first thirty minutes. The heating ramp rate was set to 20 °C min^{-1}.

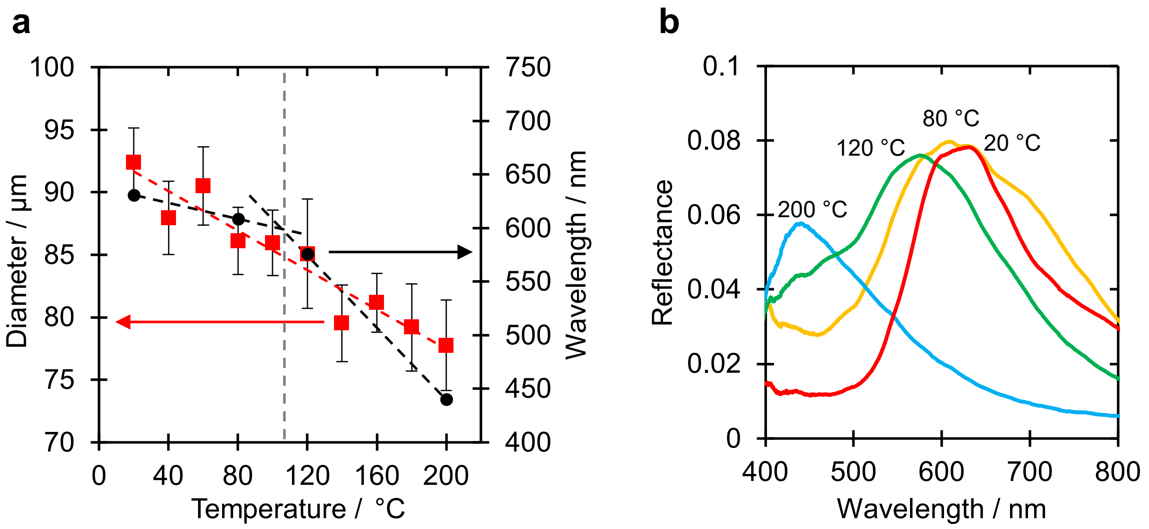

Supplementary Figure 8. (a) The linear reduction in CNC microparticle diameter upon thermal treatment (*red squares*) and the corresponding peak wavelength (*black circles*), showing the significant blueshift above 100 °C. **(b)** Representative spectra for the heat-treated CNC microparticles highlighted in (a), showing the increasing blueshift with temperature. Note that the decrease in reflected intensity at 200 °C, compared to e.g., methanol treatment (Figure 1d of the article), is attributed to the onset of thermal degradation. This is evidenced by the discolouration of the microparticles, which is apparent when imaged in the dry state (Supplementary Figure 9).

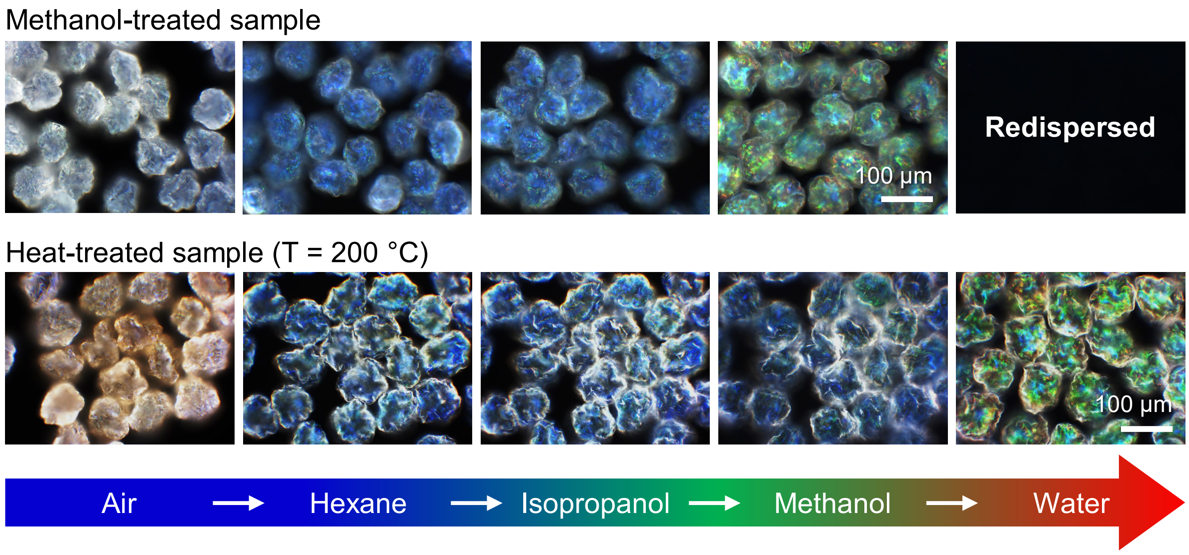

Supplementary Figure 9. Dark-field microscope images showing the responsiveness of the CNC microparticles to different solvents after post-treatment with either methanol (*top*) or by heating at 200 °C for 1h (*bottom*). Polar solvents swell and redshift the methanol-treated microparticles, with water able to completely disperse the methanol-treated sample. In contrast, the 200°C heat-treated sample did not swell as extensively and was stable in water.

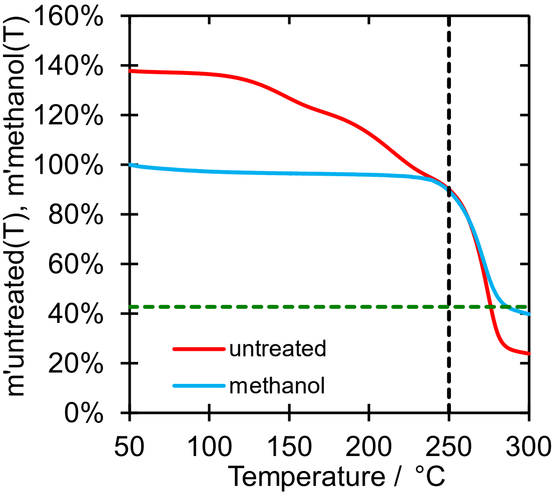

Supplementary Figure 10. Processed thermogravimetric analysis (TGA) curves of the microparticles comparing untreated microparticles (i.e., hexane-washed only, *red line*) with methanol-treated microparticles (*blue line*). The mass of the untreated sample was rescaled as $m'_{\text{untreated}}(T) = \text{TGA}_{\text{untreated}}(T)\ w_{\text{CNC},2}/w_{\text{CNC},1}$, to account for the same initial cellulose content such that it overlaps at 250 °C with the methanol-treated sample (*vertical dashed line*). This rescaling makes the residual water in the untreated microparticles more apparent, but also indicates that at 300 °C, methanol treatment significantly prevents mass loss *via* pyrolysis, approaching the theoretical maximum (*horizontal dashed line* at $42.67\% = r_{(\text{C/CNC}),\text{max}} w_{\text{CNC},2}$, see Supporting Discussion 1 for details). This agrees with the dehydrant property of methanol,[1] as the removal of water (and thus oxygen atoms) prevents mass loss *via* CO and CO_2 emission.

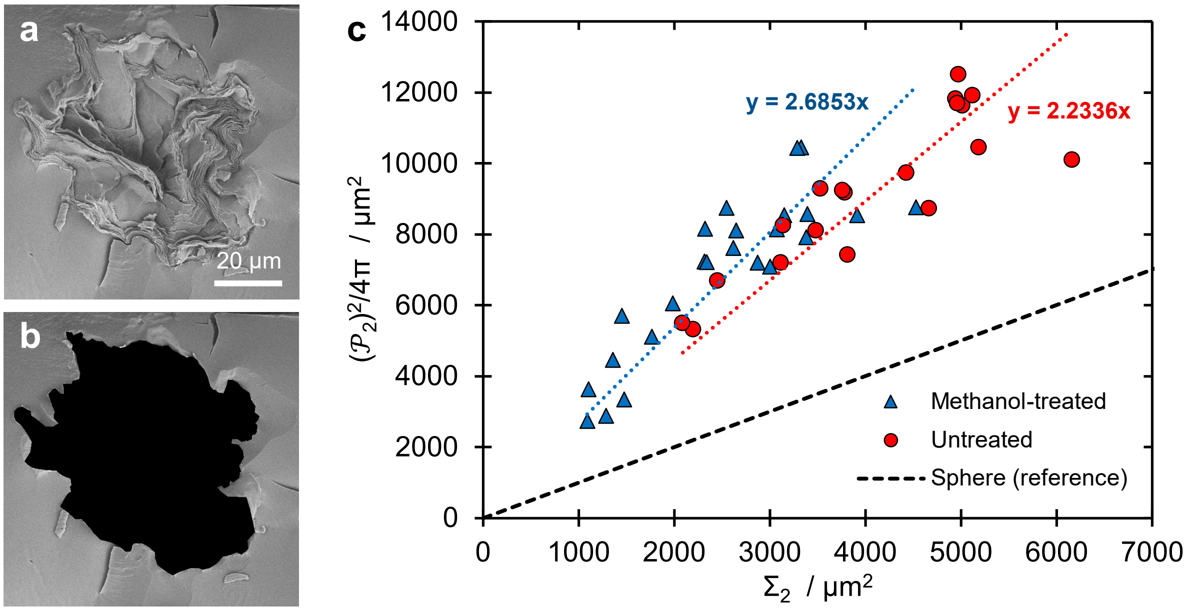

Supplementary Figure 11. (a) Example of cross-sectional SEM analysis of a methanol-treated CNC microparticle, illustrating how the buckled surface distorts the radially aligned internal helicoidal architecture. **(b)** The manually shaded area corresponding to the cross-section of the microparticle shown in (a), allowing for determination of both the apparent perimeter ($\mathcal{P}_2$) and apparent cross-sectional area (Σ_2) of the microparticle. **(c)** A plot showing the correlation between $\mathcal{P}_2$ and Σ_2 for both methanol-treated (*blue triangles*) and untreated (*red circles*) CNC microparticles with different fracture planes. The corresponding relationship for an unbuckled sphere is included for reference (*black dashed line*). The slope of the trendlines provides an estimation of β and Q, given $Q = \beta^2 = 4\pi\Sigma_2/\mathcal{P}_2^2$.

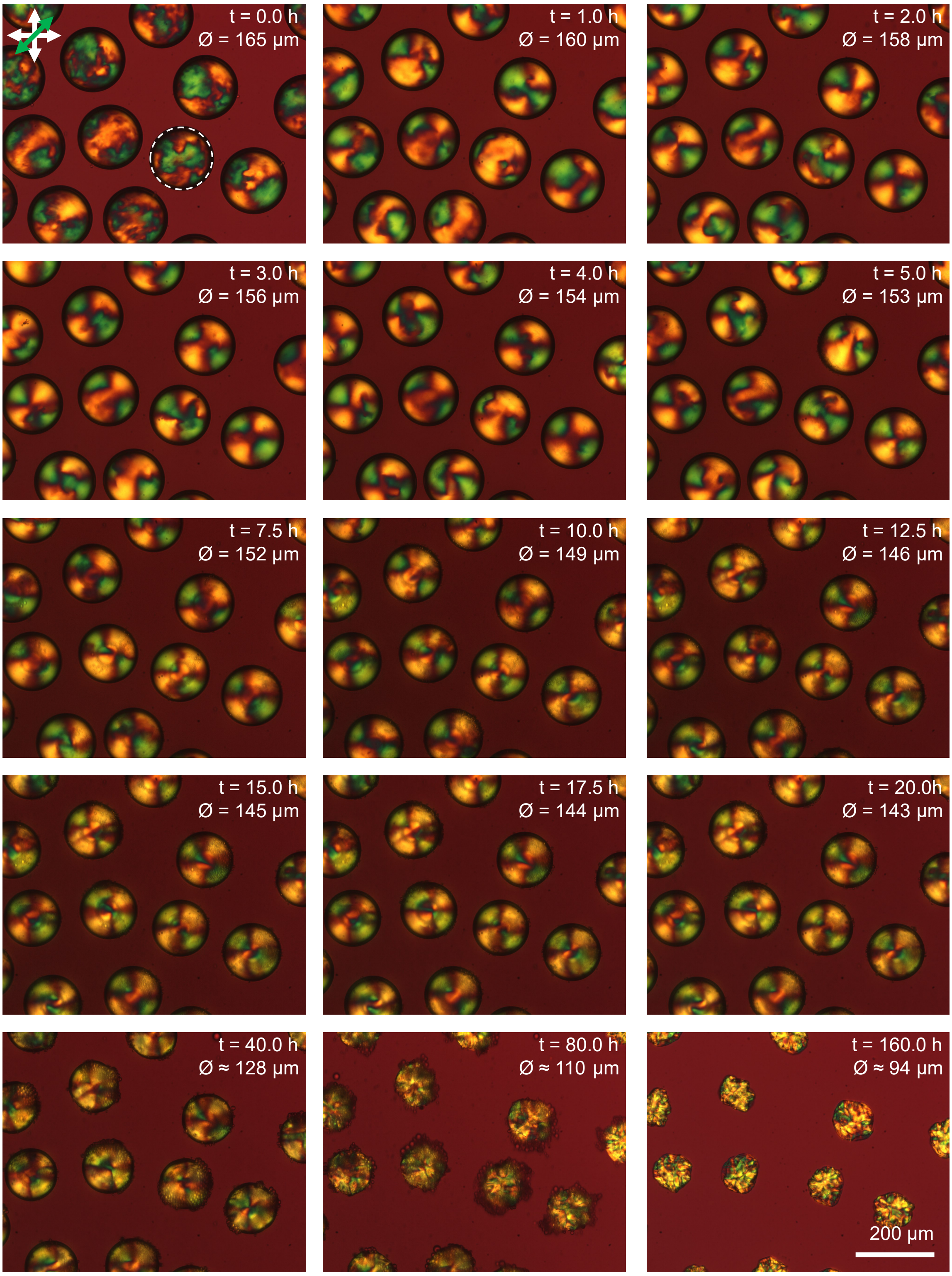

Supplementary Figure 12. Time-lapse polarised optical microscope image series showing the evolution of a drying CNC microdroplet ([CNC] = 7 wt.%, [NaCl]/[CNC] = 100 µmol g^{-1}, anisotropic phase). The micrographs were collected in transmission through crossed polarisers with a 45° full-wave retardation plate, allowing visualisation of the birefringent cholesteric phase. The yellow/blue colours indicate the mesophase orientation. The reduction in the drying rate and the cloudy appearance is due to localised saturation of the oil, resulting in surfactant-stabilised water droplets.

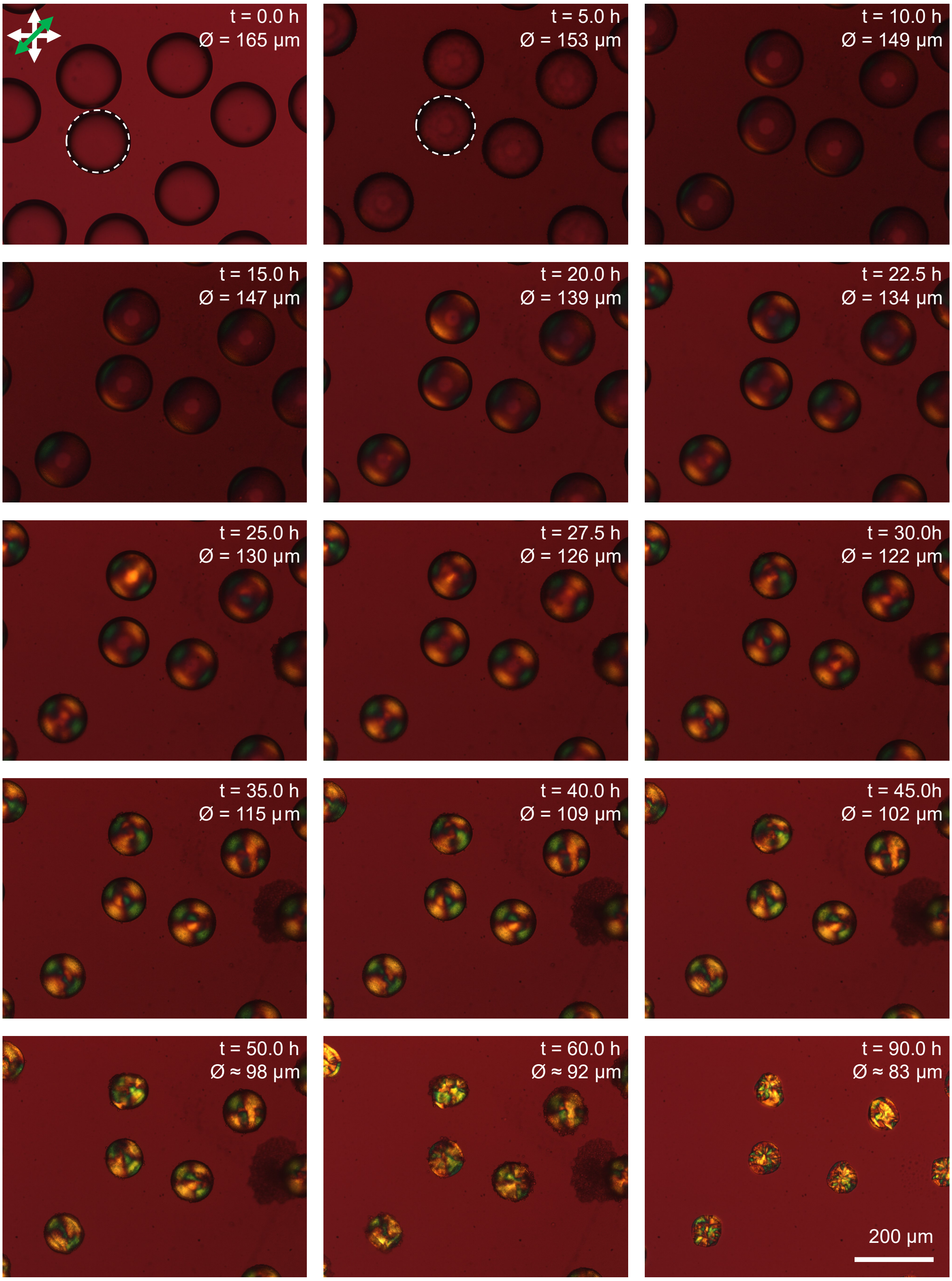

Supplementary Figure 13. Time-lapse polarised optical microscope image series showing the evolution of a drying CNC microdroplet ([CNC] = 3.5 wt.%, [NaCl]/[CNC] = 100 µmol g^{-1}, i.e., 50% dilution of the stock anisotropic suspension). The micrographs were collected in transmission through crossed polarisers with a 45° full-wave retardation plate, allowing visualisation of the birefringent cholesteric phase. The yellow/blue colours indicate the mesophase orientation. The reduction in the drying rate and the cloudy appearance is due to localised saturation of the oil, resulting in surfactant-stabilised water droplets.

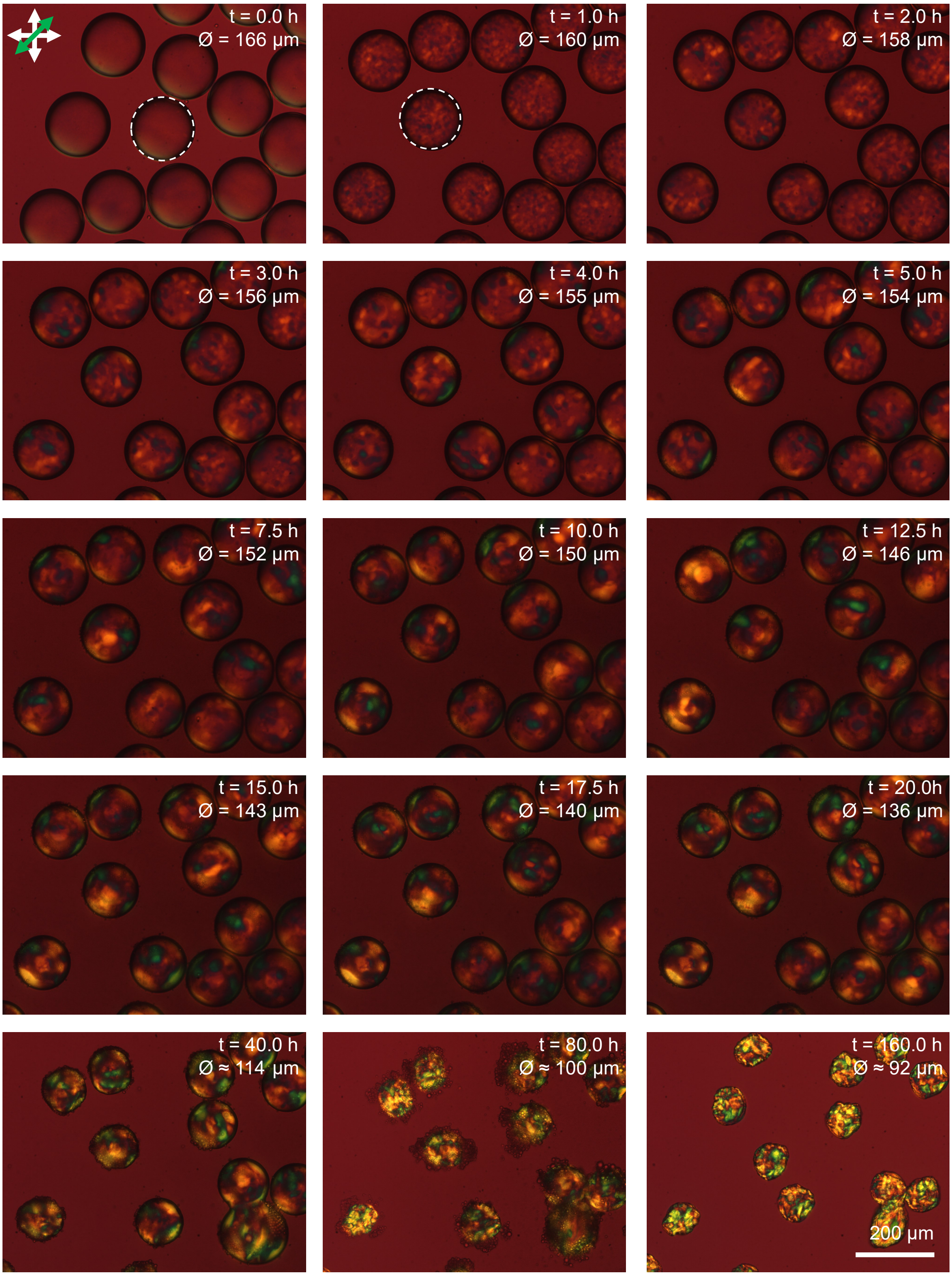

Supplementary Figure 14. Time-lapse polarised optical microscope image series showing the evolution of a drying CNC microdroplet ([CNC] = 7 wt.%, [NaCl]/[CNC] = 100 μmol g^{-1}, isotropic phase). The micrographs were collected in transmission through crossed polarisers with a 45° full-wave retardation plate, allowing visualisation of the birefringent cholesteric phase. The yellow/blue colours indicate the mesophase orientation. The reduction in the drying rate and the cloudy appearance is due to localised saturation of the oil, resulting in surfactant-stabilised water droplets.

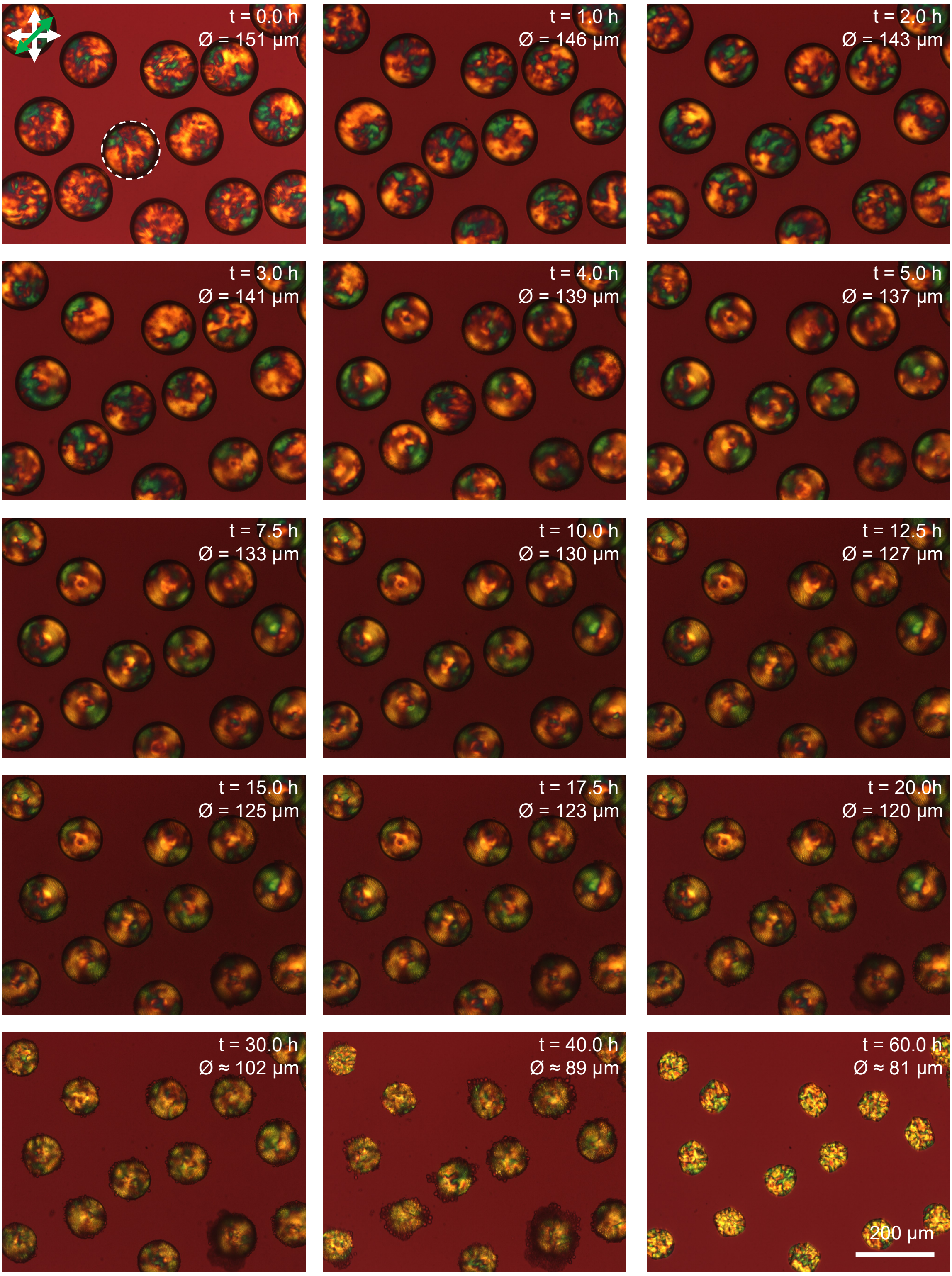

Supplementary Figure 15. Time-lapse polarised optical microscope image series showing the evolution of a drying CNC microdroplet ([CNC] = 7.0 wt.%, [NaCl]/[CNC] = 0 μmol g^{-1}, i.e., no added salt). The micrographs were collected in transmission through crossed polarisers with a 45° full-wave retardation plate, allowing visualisation of the birefringent cholesteric phase. The yellow/blue colours indicate the mesophase orientation. The reduction in the drying rate and the cloudy appearance is due to localised saturation of the oil, resulting in surfactant-stabilised water droplets.

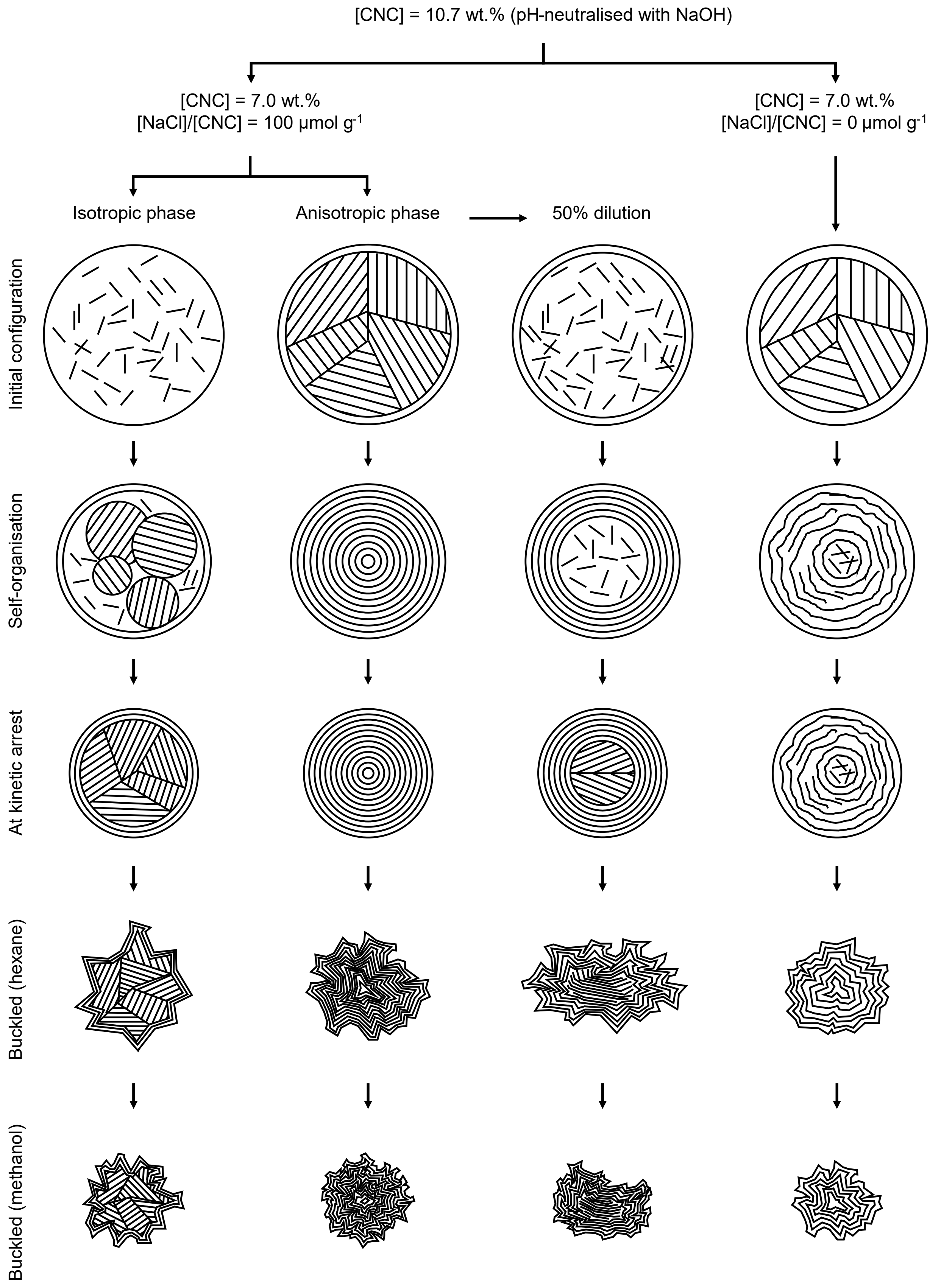

Supplementary Figure 16. Schematic describing the self-assembly pathways for the different formulations of the CNC suspension, including the effect of solvent desiccation as a post-treatment. The morphology is derived from the time-lapse studies reported in Supplementary Figures 12-15 and the optical and electron microscopy in Figure 6 of the article.

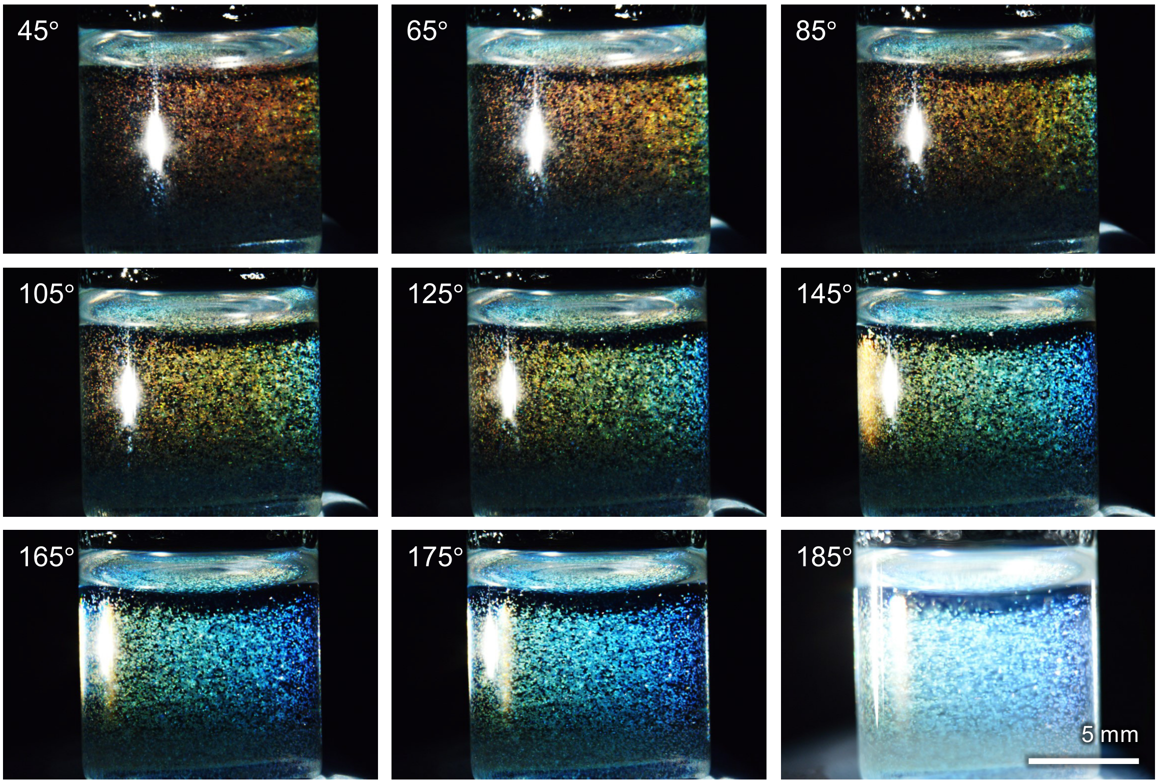

Supplementary Figure 17. Photograph sequence showing the iridescence of red CNC microparticles dispersed in refractive index matching oil ($n = 1.55 \approx n_{\text{CNC}}$) within a cylindrical glass vial, when viewed at 0° and with the illumination angle of a directed white light beam (3300 K) increased from α_i = 45° to 180° (as defined with respect to the viewing direction). This geometry allows the incident and outgoing light beams inside the suspension to remain relatively unaffected by Snell's law at the air/glass and glass/oil interfaces. In this configuration, the particles showed a drastic colour change from red to blue with increasing α_i.

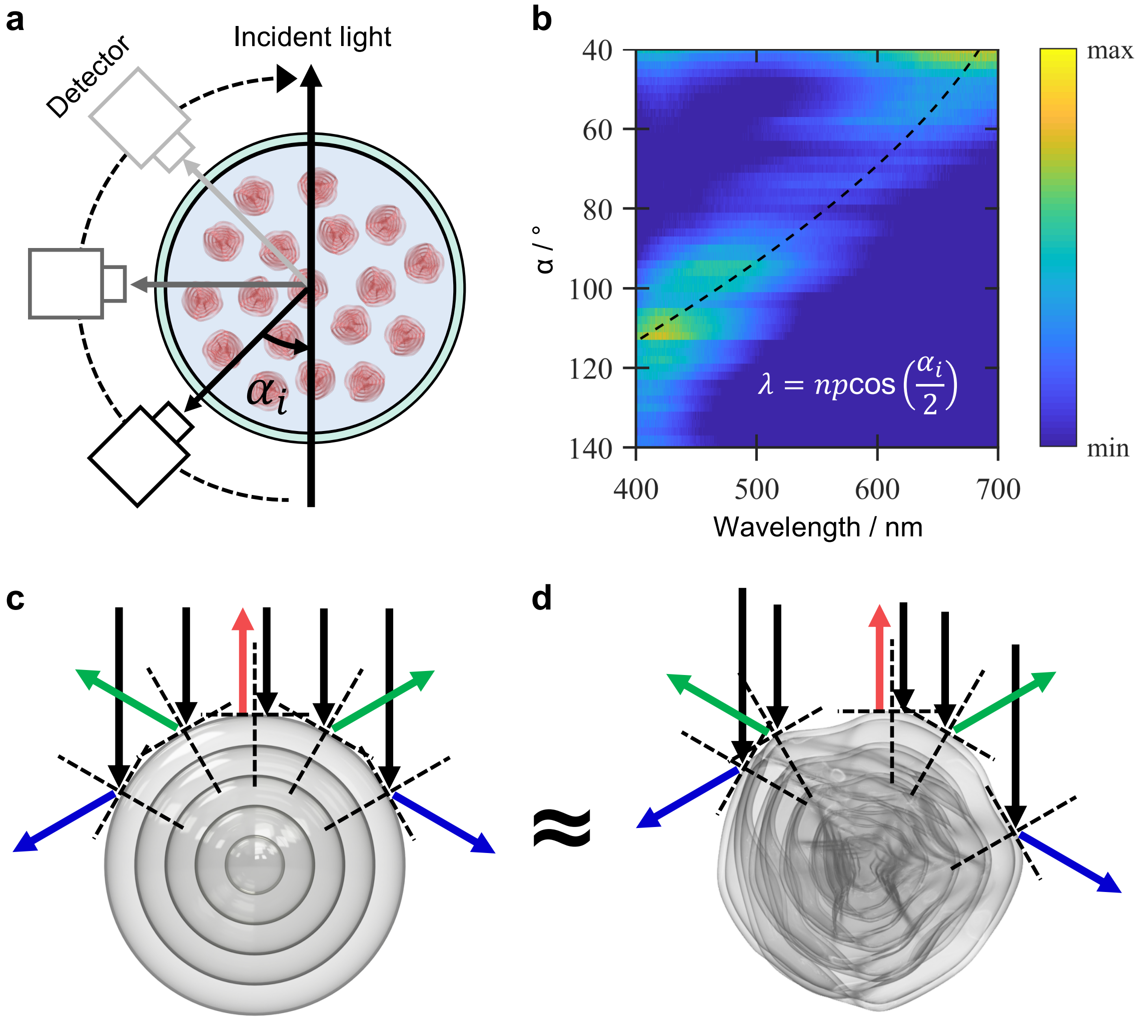

Supplementary Figure 18. (a,b) Angular resolved spectrometry of the red CNC microparticles dispersed in refractive index matching oil ($n = 1.55 \approx n_{\mathrm{CNC}}$) within a cylindrical glass vial (see Supplementary Figure 17), where the sample and illumination direction are fixed, and the detector is swept over half a turn (equivalent to α_i = 45° to 180°). The peak reflection shifts according to Bragg's law and as such the microparticles can be considered to be always at specular conditions. This optical behaviour matches surprisingly well with the expected isotropic response for a radially aligned cholesteric sphere,[5,6] despite the presence of buckling. This confirms that buckling has minimal impact on the macroscopic angular optical response of the CNC microparticles. **(c,d)** A simplistic model explaining the similarity between the optical response of (c) the non-buckled microparticles and (d) buckled microparticles under these illumination conditions.

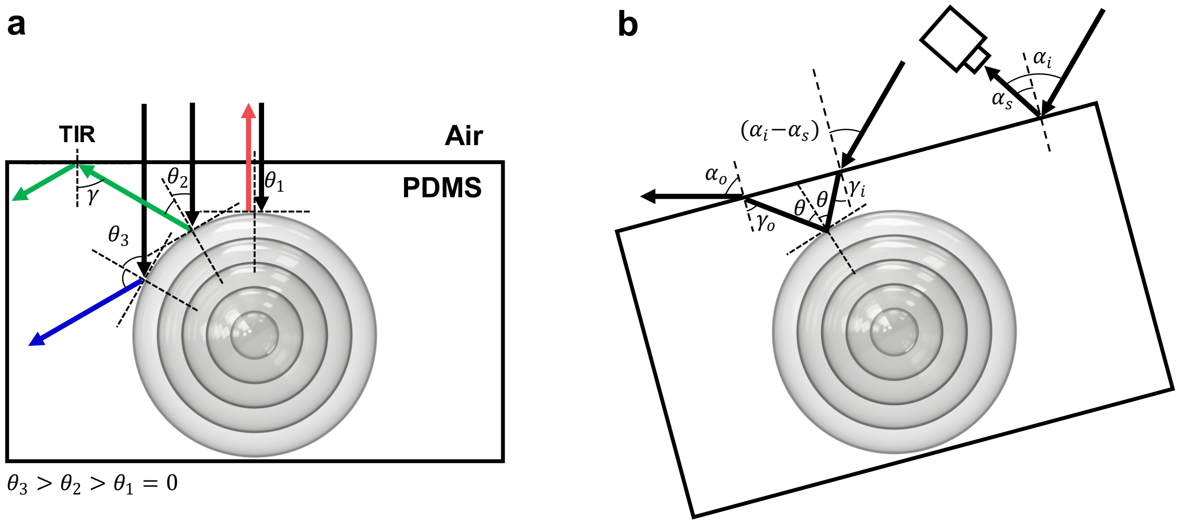

Supplementary Figure 19. (a) Schematic illustrating the conditions required for light reflected from an embedded CNC microparticle to escape from the PDMS matrix, for the simple case where the incident light is perpendicular to the air/film interface. **(b)** A more complicated situation where the sample angles and illumination angles are at arbitrary values.

When considering broadband incident light perpendicular to the film surface, as in (a), refraction at the resin-air interface does not occur. Among the beams that impinge a given microsphere, those with incident angle $\theta > 45°$ are reflected towards the back of the film (e.g., blue arrow). Light with incident angle $\theta < 45°$ arrives at the resin-air interface at an angle $\gamma = 2\theta$. If this angle γ is larger than the critical angle ($\sim 44.3°$ for PDMS resin with n_{PDMS} = 1.43), total internal reflection occurs according to Snell's law and the light cannot escape the resin (e.g., green arrow). Therefore, the maximum θ = $44.3°/\ 2$ = 22.1°. The light incident on to the CNC microsphere will refract further to reduce the incident angle ($20.3°$), making the ratio between the detectable minimum and maximum wavelength: $\cos(20.3°) \approx 0.94$, which approximates to a maximum wavelength shift of 30 nm for the green CNC photonic pigment. In the more complex case, as illustrated in (b), the relationship between different angles are as following:

$$\gamma_i = \arcsin\left(\frac{1}{n_{\text{PDMS}}}\sin\left(\alpha_i - \alpha_s\right)\right)$$

θ: variables - anything between (0, 90), determining the colour by $\lambda = np\ \cos\left(\text{arcsin}\left(\frac{n_{\text{PDMS}}}{n}\ \sin\theta\right)\right)$

$$\gamma_o = 2\theta - r_i$$

$$\alpha_o = \arcsin(n\sin(\gamma_o))$$

where $n = n_{\text{CNC}} = 1.55$, α_i is the angle between the incident light and the film surface, α_s is the angle between detection and film surface, and α_o is the angle between the refracted light beam and the film surface.

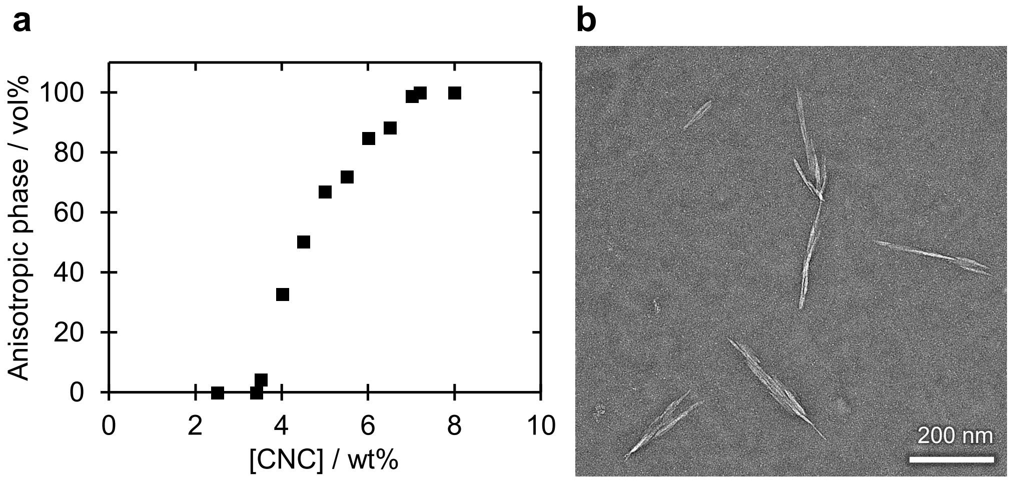

Supplementary Figure 20. (a) Volumetric ratio of anisotropic phase as a function of the concentration of the commercial CNC suspension (i.e., no added salt), as extracted from photographs of glass capillaries between crossed polarisers. **(b)** Transmission electron micrograph (TEM) of individual CNCs, showing their elongated, splinter-like morphology.

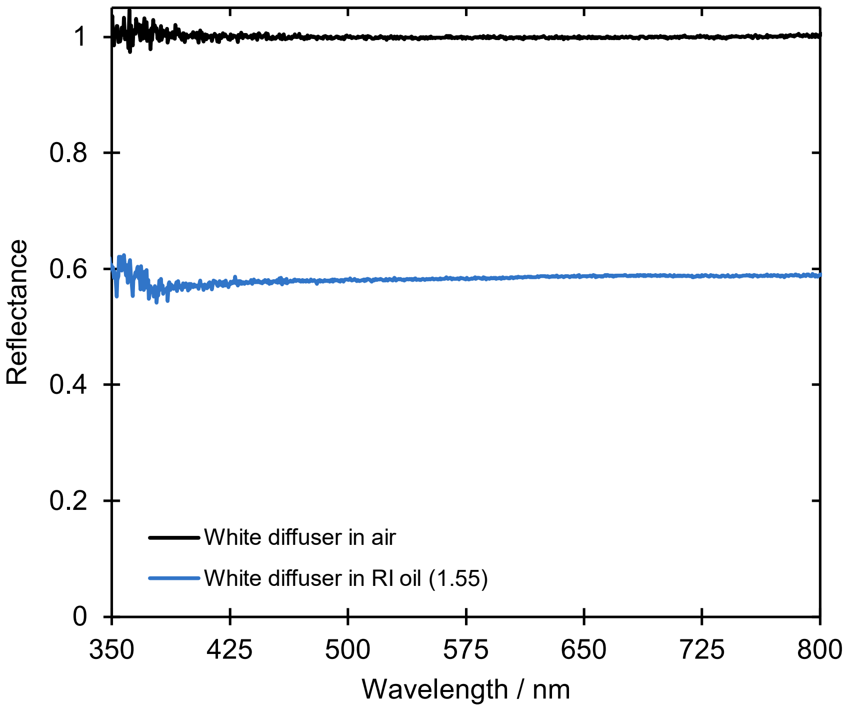

Supplementary Figure 21. Comparison of the reflectance from a white diffuser in dark-field illumination either (i) in air (*black line*) or (ii) coated with a thin droplet of refractive index oil (n = 1.55, *blue line*), both spectra are normalised against the same white diffuser in air. The microscope configuration is the same as that used to analyse the CNC microparticles. By referencing the optical response of the CNC microparticles against a white diffuser coated with oil the specular reflection from the air-oil interface can be negated. However, it should be noted that as the optical response of the CNC microparticles is not based upon scattering, but arises from a distorted, radially aligned cholesteric ordering, the spectra reported in Figure 1 of the article may still underrepresent the true reflectivity of the pigments.[7]

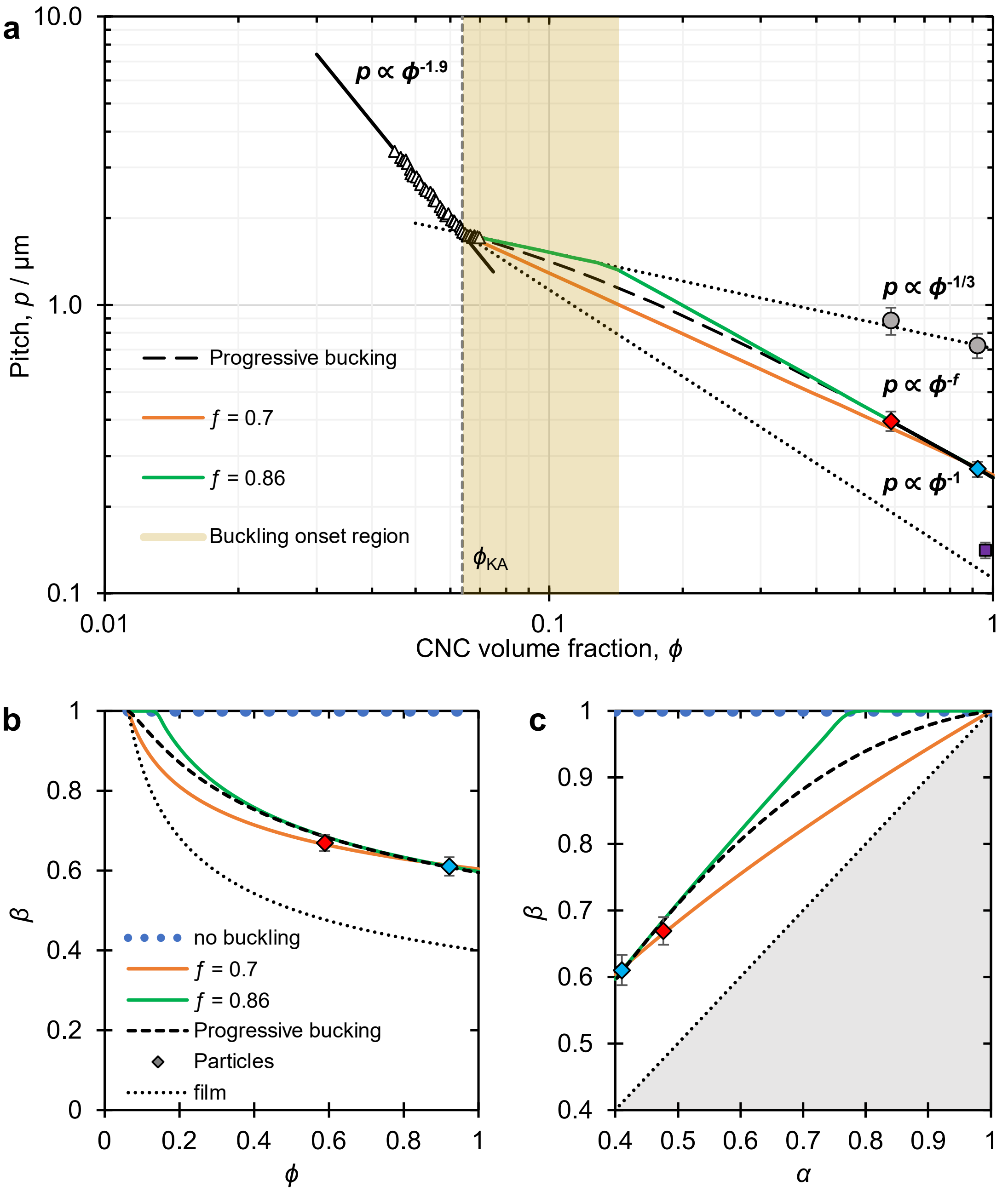

Supplementary Figure 22. (a) Pitch diagram describing the different compression scenarios after the onset of kinetic arrest: (i) vertical domain in a planar geometry, (ii) no buckling in a spherical geometry, (iii) power law ϕ^{-f} with f = 0.7 when assuming a power law between β and α, (iv) delayed buckling with an initial unbuckling behaviour, followed by a power law f = 0.86, assuming a power law between β and α, (v), a progressive buckling occurring at the onset of kinetic arrest and developing at higher volume fractions. The onset of buckling must be between the extremes of iii and iv, as denoted by the brown region. Coloured diamonds represent the particle pitch (from SEM) vs volume fraction (from TGA). Error bars represent the average deviation of p_{limb} from SEM cross sections **(b)** Scaling laws between β and ϕ for the different scenarios, and **(c)** the corresponding scaling law between β and α (with the shaded triangle corresponding to unphysical β values outside the [α, 1] range). In (b) and (c) the coloured diamonds represent β, as estimated using Q from the SEM analysis, while error bars represent the uncertainty of the mean β values and were estimated from propagating p_{limb} and Q uncertainties.

References

1. Kim, D. Y., Nishiyama, Y., Wada, M. & Kuga, S. High-yield carbonization of cellulose by sulfuric acid impregnation. *Cellulose* **8**, 29–33 (2001).
2. Frka-Petesic, B., Kamita, G., Guidetti, G. & Vignolini, S. Angular optical response of cellulose nanocrystal films explained by the distortion of the arrested suspension upon drying. *Phys. Rev. Mater.* **3**, 045601 (2019).
3. Frka-Petesic, B., Guidetti, G., Kamita, G. & Vignolini, S. Controlling the Photonic Properties of Cholesteric Cellulose Nanocrystal Films with Magnets. *Adv. Mater.* **29**, 1701469 (2017).
4. Parker, R. M. *et al.* Hierarchical Self-Assembly of Cellulose Nanocrystals in a Confined Geometry. *ACS Nano* **10**, 8443–8449 (2016).
5. Geng, Y., Noh, J. H., Drevensek-Olenik, I., Rupp, R. & Lagerwall, J. Elucidating the fine details of cholesteric liquid crystal shell reflection patterns. *Liq. Cryst.* **44**, 1948–1959 (2017).
6. Noh, J., Liang, H. L., Drevensek-Olenik, I. & Lagerwall, J. P. F. Tuneable multicoloured patterns from photonic cross-communication between cholesteric liquid crystal droplets. *J. Mater. Chem. C* **2**, 806–810 (2014).
7. Chan, C. L. C. *et al.* Visual Appearance of Chiral Nematic Cellulose-Based Photonic Films: Angular and Polarization Independent Color Response with a Twist. *Adv. Mater.* **31**, 1905151 (2019).
