# Supplementary material for: Cellulose photonic pigments
Source: Nat Commun. 2022 Jun 13;13:3378. doi: 10.1038/s41467-022-31079-9 (PMC9192732; doi:10.1038/s41467-022-31079-9)
Supplement: Supplementary file 2 — Description of Additional Supplementary Files [file 41467_2022_31079_MOESM2_ESM.pdf]

Description of Additional Supplementary Files

File Name: Supplementary Movie 1
Description: A timelapse of CNC microdroplets drying to form photonic microparticles.
